# Genomic Insights into the Radiation-Resistant Capability of *Sphingomonas qomolangmaensis* S5-59^T^ and *Sphingomonas glaciei* S8-45^T^, Two Novel Bacteria from the North Slope of Mount Everest

**DOI:** 10.3390/microorganisms10102037

**Published:** 2022-10-14

**Authors:** Yang Liu, Xiaowen Cui, Ruiqi Yang, Yiyang Zhang, Yeteng Xu, Guangxiu Liu, Binglin Zhang, Jinxiu Wang, Xinyue Wang, Wei Zhang, Tuo Chen, Gaosen Zhang

**Affiliations:** 1 State Key Laboratory of Cryospheric Sciences, Northwest Institute of Eco-Environment and Resources, Chinese Academy of Sciences, Lanzhou 730000, China; 2 University of Chinese Academy of Sciences, No. 19A Yuquan Road, Beijing 100049, China; 3 Key Laboratory of Extreme Environmental Microbial Resources and Engineering, Lanzhou 730000, China; 4 Key Laboratory of Desert and Desertification, Northwest Institute of Eco-Environment and Resources, Chinese Academy of Sciences, Lanzhou 730000, China; 5 College of Geography and Environment Science, Northwest Normal University, Lanzhou 730070, China; 6 College of Urban Environment, Lanzhou City University, Lanzhou 730070, China; 7 School of Stomatology, Lanzhou University, Lanzhou 730000, China

**Keywords:** moraine, Mount Everest, extremophiles, whole-genome sequencing, DNA repair, radiation resistance

## Abstract

Mount Everest provides natural advantages to finding radiation-resistant extremophiles that are functionally mechanistic and possess commercial significance. (1) Background: Two bacterial strains, designated S5-59T and S8-45T, were isolated from moraine samples collected from the north slope of Mount Everest at altitudes of 5700m and 5100m above sea level. (2) Methods: The present study investigated the polyphasic features and genomic characteristics of S5-59^T^ and S8-45^T^. (3) Results: The major fatty acids and the predominant respiratory menaquinone of S5-59^T^ and S8-45^T^ were summed as feature 3 (comprising C16:1 *ω*6c and/or C16:1 *ω*7c) and ubiquinone-10 (Q-10). Phylogenetic analyses based on 16S rRNA sequences and average nucleotide identity values among these two strains and their reference type strains were below the species demarcation thresholds of 98.65% and 95%. Strains S5-59^T^ and S8-45^T^ harbored great radiation resistance. The genomic analyses showed that DNA damage repair genes, such as *mutL*, *mutS*, *radA*, *radC*, *recF*, *recN*, etc., were present in the S5-59^T^ and S8-45^T^ strains. Additionally, strain S5-59^T^ possessed more genes related to DNA protection proteins. The pan-genome analysis and horizontal gene transfers revealed that strains of *Sphingomonas* had a consistently homologous genetic evolutionary radiation resistance. Moreover, enzymatic antioxidative proteins also served critical roles in converting ROS into harmless molecules that resulted in resistance to radiation. Further, pigments and carotenoids such as zeaxanthin and alkylresorcinols of the non-enzymatic antioxidative system were also predicted to protect them from radiation. (4) Conclusions: Type strains S5-59^T^ (=JCM 35564T =GDMCC 1.3193T) and S8-45^T^ (=JCM 34749T =GDMCC 1.2715T) represent two novel species of the genus *Sphingomonas* with the proposed name *Sphingomonas qomolangmaensis* sp. nov. and *Sphingomonas glaciei* sp. nov. The type strains, S5-59^T^ and S8-45^T^, were assessed in a deeply genomic study of their radiation-resistant mechanisms and this thus resulted in a further understanding of their greater potential application for the development of anti-radiation protective drugs.

## 1. Introduction

Mount Everest (which is also referred to as Mount Qomolangma in China) is the world’s highest mountain. It has extremely harsh ecological niches, including strong radiation, low precipitation, low temperature, deep-frozen soil, low concentration of atmospheric oxygen, etc. [1,2]. Generally, such extreme habitats are considered to be uninhabitable by most lifeforms [3]. However, some studies have reported that the polar regions’ (comprising the Antarctic, Arctic, and Tibetan Plateau) soils harbor distinct survival patterns of microbial diversity and exhibit dominant, special functional phylotypes that adapt to harsh conditions [4]. For instance, Yang et al. investigated the *Planococcus* halotolerant Y50^T^ that was isolated from the Tibet Plateau, which possessed the ability of petroleum degradation, as well as antioxidant, radiation resistance, and cold adaptation [5]; further, *Lentzea tibetensis* FXJ1.1311^T^ was isolated from the soil of the Tibetan Plateau, which showed antimicrobial activity against Gram-positive bacteria and Fusarium oxysporum [6]. Additionally, more novel microbial resources with special functions formed by adaptation to such extreme environments could survive here [3,7,8,9]. As we all know, the high-altitude Mount Everest in the Tibetan Plateau region of China has currently been receiving a large amount of solar radiation—and the higher the altitude, the more cosmic rays it receives [3,10]. Previous studies have reported that a large number of bacteria with radiation resistance live in the moraine soils of the glacier at high altitudes; further, they also have strong antioxidant and cold resistance functions, as well as other functions to cope with the extreme conditions [8,11,12]. However, there are fewer reports of novel functional extremophiles in the less-interfered regions of humans and in the higher altitude of the northern slopes of the Mount Everest region, especially on radiation-resistant microorganisms. Therefore, the exploitation of microbial resources that survive in these extreme ecological niches with high doses of radiation provides an important basis for an in-depth study of their radiation-resistant mechanism and the industrial potential for the development of anti-radiation protective agents.

Recently, some studies reported that radiation, including ionizing radiation (IR) and non-ionizing radiation (NIR), could affect cellular biomolecules–including nucleic acids, proteins, and lipids–directly or indirectly [13,14]. For instance, IR such as cosmic rays (consisting of γ-rays, α-particles, neutrons, etc.) in nature could disrupt the DNA structure directly through the damage of the sugar backbone and the purine/pyrimidine base [15]. Moreover, the reactive oxygen species (ROS), which include hydroxyl radicals (OH•), super-oxide anions (O^2−^), hydrogen peroxide (H_2_O_2_), etc., could damage—by radiolysis of H_2_O in the process of the IR and NIR (UV) radiation—nucleic acids, carbohydrates, proteins, and lipids indirectly [14,15,16]. DNA single-strand breaks (SSBs) or double-strand breaks (DSBs) are the result of significant lethal damage to the chromosomal DNA of living microorganisms under radiation-induced conditions, followed by oxidative damage to cells from the accumulation of ROS generated by irradiation [14,17,18]. When microorganisms face radiation damage, their DNA repair mechanisms, reactive oxygen species (ROS) detoxification mechanisms of enzymatic and non-enzymatic antioxidative systems, induced protein folding and degradation systems, and also their accumulation of compatible solute models will respond to resist or repair radiation damage [14,19]. Most of the previous studies on these mechanisms of microbial resistance to radiation have focused on those microorganisms that can resist the extreme doses of irradiation of up to a few thousand with survival acute doses higher than 1 KGy (1Gy = 100 rad), and even to 15 KGy [20,21,22]. However, Daly and Kenneth suggest that a human exposed to less than 5 Gy of ionizing radiation would suffer almost certain death [23], and what is more important is that the chronic accumulation of radiation will also produce irreversible damage to living organisms [24]. Therefore, it is important to study the mechanisms involved when applying radiation in a micro-dose, as well as the mechanisms of chronic irradiation damage when using radiation-resistant microorganisms that have been subjected to long-term irradiation stress in natural environments. Meanwhile, the distribution of radiation-resistant microorganisms has specific ecological niches due to non-intensive levels of ionizing radiation in natural environments, and the response mechanisms of microorganisms to radiation could be different for different radiation-stressed ecological niches [14,25]. Therefore, radiation-resistant microorganisms, screened in long-term highly intensive irradiated natural habitats, such as Mount Everest, will enable one to obtain a better insight into the understanding of radiation-resistant molecular mechanisms.

The genus *Sphingomonas* was assigned to the phylum Proteobacteria and the first description of the original member of this genus, *Sphingomonas paucimobilis*, was detailed by Yabuuchi et al. [26]. Up to now, 171 species of the genus *Sphingomonas* have been recognized with validated, published names (https://lpsn.dsmz.de/genus/sphingomonas accessed on 12 February 2022). According to previous research by others, species of the genus *Sphingomonas* can be found distributed throughout ecological niches of extreme environments, such as the soil of subterranean sediment [27], desert [28], glacier ice [29], moraine [11], etc. Sajjad et al. systematically reported the important role of the *Sphingomonas* species in polycyclic aromatic hydrocarbon (PAH) degradation, plant growth promotion, stress tolerance, and in many other important traits [30]. However, limited studies are available on the *Sphingomonas* species’ associated radiation resistance. Although some studies have reported novel *Sphingomonas* species’ radiation resistance [11,31], the genomic insight on radiation resistance mechanisms in *Sphingomonas* has rarely been discussed. Here, we reported two novel radiation-resistant species, *S. qomolangmaensis* S5-59^T^ and *S. glaciei* S8-45^T^, isolated from the high altitude of 5700m a.s.l. (above sea level) and 5100m a.s.l., respectively, in the north slope area of Mount Everest where high doses of irradiation and other extremely harsh conditions occur all year round. Meanwhile, exploration and research of the genus *Sphingomonas* in radiation-resistant mechanisms are rare, which has also prompted us to conduct in-depth whole-genome sequencing analysis for these two novel species, which are found in ecological niches at different altitudes. Therefore, in this study, we performed whole-genome sequencings of *S. qomolangmaensis* S5-59^T^ and *S. glaciei* S8-45^T^ in order to reveal the radiation-resistant mechanisms of the genus *Sphingomonas* that are found in glacial environments and with high-doses of irradiation.

## 2. Materials and Methods

### 2.1. Bacteria Isolation and Growth Conditions

Strains S5-59^T^ and S8-45^T^ were isolated from the moraine soil samples that were collected on 8 May 2019 at the high altitude of 5700m above sea level (a.s.l.) and 5100m a.s.l., respectively, in the north slope area of Mount Everest (28.02° N, 86.56° E)—which is in the Tibetan Plateau region of China near East Rongbuk glacier, and which has an extremely harsh ecological niche with high ultraviolet and cosmic ray radiation, low temperature, and low concentration of atmospheric oxygen [1]. When conducting the enrichment experiments for isolating the strains S5-59^T^ and S8-45^T^, 5 g of the moraine soil sample was enriched in 20 mL sterile saline (0.85%) for 4h at 30 °C, shaken at 200 rpm. Then, the enriched solution was diluted and spread on Reasoner’s 2A (R2A) agar medium [32] and incubated at 30 °C for 15 days. Subsequently, strains S5-59^T^ and S8-45^T^ were purified in the R2A agar medium for further analysis. The reference type strains *S. panacisoli* HKS19^T^ were purchased from Belgian Coordinated Collections of Microorganisms (BCCM); *S. asaccharolytica* DSM 10564^T^ and *S. panacis* DCY99^T^ were purchased from the Japan Collection of Microorganisms (JCM); and *S. kaistensis* PB56^T^, *S. astaxanthinifaciens* DSM 22298^T^, and *S. ginsengisoli* KCTC 12630^T^ were purchased from the German Collection of Microorganisms and Cell Cultures and Cell Cultures GmbH (DSMZ).

### 2.2. Morphological, Physiological, and Biochemical Analysis

The morphological characteristics of strains S5-59^T^ and S8-45^T^ were observed after 72 h of incubation in the R2A agar medium. The Gram reaction was tested by a Solarbio Gram staining kit (Solarbio Cat#G1132, Beijing, China). The size and morphology of cells were observed using electron microscopy (JSM-5600, JEOL). Growth temperature tests were performed on the R2A liquid medium in the range of 5–50 °C at intervals of 5 °C. NaCl resistance tests were performed on the R2A liquid medium containing 0–10% (*w*/*v*) at intervals of 1%. The growth pH range was determined using R2A liquid media with pH 4–12 at intervals of 1. The carbohydrate utilization test, nitrogen utilization test, and hydrolysis tests were determined according to the methods of Shirling and Gottlieb, Williams, and Kurup and Schmitt, respectively [33,34,35]. Other enzyme activities were detected by using API ZYM strips according to the manufacturer’s instructions (biomé Rieux, Lyon, France).

### 2.3. Chemotaxonomic Analysis

For the analysis of the chemotaxonomic features of strains S5-59^T^ and S8-45^T^—as well as their closely related type strains, *S. panacisoli* HKS19^T^; *S. asaccharolytica* DSM 10564^T^; *S. panacis* DCY99^T^; *S. kaistensis* PB56^T^; *S. astaxanthinifaciens* DSM 22298^T^; and *S. ginsengisoli* KCTC 12630^T^—a series of experiments were carried out to determine the content of the respiratory quinones, polar lipids, and fatty acids with cell biomass obtained from cultures grown in R2A agar for 72 h at 30 °C. Respiratory quinones were extracted (<37 °C) from dried organisms (100 mg) with chloroform/methanol (2:1, *v*/*v*) and were analyzed by the HPLC system [36]. Diaminoacrylic acid isomers of the cell wall and whole-cell sugars were analyzed by the methods described by Lechevalier and Lecheyalier [37] and also Staneck and Roberts [38]. Detecting the polar lipids extracted by the chloroform/methanol/water system via two-dimensional TLC, identification was performed according to the description of Minnikin et al. [39]. Cellular fatty acid methyl esters were saponified, methylated, and extracted as per the method described by Sasser [40]. Further, they were then tested and analyzed using the standard protocol of Sherlock MIDI (microbial identification system 6.2b). Peak results were determined via comparison with the database TSBA 6 (version 6.21).

### 2.4. Phylogenetic Analysis

The 16S rRNA gene sequencing was identified via polymerase chain reaction (PCR) with universal primers 27F (5′-AGAGTTTGATCCTGGCTCAG-3′) and 1492R (5′-CGGTTACCTTGTTACGACTT-3′) [41]. The PCR product was sequenced by the Tsingke Company (Xian, PR China) using the dideoxy chain termination method with an ABI 3730XL Analyzer (Applied Biosystems), and the complete gene sequencing of the 16S rRNA was complied with SeqMan software (Lasergene). For the 16S rRNA gene sequences EzBioCloud’s Identify services were used to obtain sequence information. The 16S rRNA gene sequencings were aligned using ClustalW [42]; then, the phylogenetic trees—based on 16S rRNA by the MEGA 11 [43] software package using the neighbor-joining (NJ) [44], minimum-evolution, and maximum-likelihood (ML) methods followed by bootstrap analysis with 1000 bootstrap resamplings [45]—were reconstructed. Kimura’s two-parameter model [46] was used as the model for estimating genetic differences of nucleotide substitution. The phylogenomic tree was reconstructed based on the up-to-date bacterial core gene set (UBCG), according to the pipeline suggested by Na et al. [47].

### 2.5. Genome Sequencing, Assembly, Annotation, and Comparative Genomic Analysis

The genomic DNA of strains S5-59^T^ and S8-45^T^ were extracted by a bacterial genomic DNA extraction kit (OMEGA) and sequenced by an Illumina Hiseq 2000 platform. High-quality data sets were generated for analysis, and the corresponding sequencing depths were 100X. The bacterial genome scanning maps were completed by using the short sequence assembly software SOAPdenovo2 [48], and the bacterial genome completion maps were assembled by using the assembly software unicycler V0.4.8 [49]. Glimmer was used to predict the assembly results of the scanning maps, and the chromosome genome. The average nucleotide identity (ANI) was calculated using the OrthoANIu (OrthoANI), BLAST (ANIb), and MUMmer (ANIm) algorithms [50,51,52,53]. The average amino acid identity (AAI) was calculated using the online resource from the Konstantinidis group (http://enve-omics.ce.gatech.edu/aai/ accessed on 5 January 2022) [54]. The dDDH results were calculated by using GGDC2.0 (genome-to-genome distance calculator) [55]. The dDDH results were obtained from the recommended formula 2, which was independent of genome length and robust against the utilization of incomplete draft genomes.

The tRNA genes, rRNA genes, and noncoding rRNA genes were predicted by the NCBI Prokaryotic Genome Annotation Pipeline (PGAP) [56]. The circular RNAs were detected by the northern blotting protocol [57]. Rapid Annotation of Subsystem Technology (RAST) was used to annotate the genomes of strains S5-59^T^ and S8-45^T^ [58]. For assigning or improving general functional annotations, KEGG (Kyoto Encyclopedia of Genes and Genomes) [59], COG (clusters of orthologous groups of proteins) [60], NR (NCBI non-redundant protein) [61], Pfam (protein families) [62], Swiss-Prot [63] and CAZy (carbohydrate active enzymes) databases were selected for retrieval [64]. AntiSMASH 6.0.1 was used to in silico predict the biosynthetic gene clusters of secondary metabolites (https://antismash.secondarymetabolites.org/ accessed on 10 January 2022) [65]. The statistical analyses were performed using SPSS16.0 software and R 4.1.0 software. The pan genome was constructed using the Bacterial Pan Genome Analysis (BPGA) software [66]. The genome sequencings of strains S5-59^T^ and S8-45^T^ were deposited in the GenBank database with accession numbers CP101740 and CP097253, respectively.

### 2.6. The Radiation Resistance Analysis

The assay of radiation-resistant ability was detected using ionizing (γ-rays) and non-ionizing (UVC) radiation [11]. The strains S5-59^T^, S8-45^T^, and reference type strains were inoculated into 100 mL R2A liquid media, shaken at 200 rpm, cultured at 30 °C for 72 h, and the cell suspensions were then collected. The reference type strain Escherichia coli BL 21 was the negative control. The cell suspensions of all strains were adjusted to OD600 = 1 and then divided into three aliquots. One aliquot was serially diluted and coated on an R2A agar medium, and a portion of the plate was stored as the control without radiation, while the rest was exposed to UVC irradiation at different doses of 20, 50, 100, and 150 J/m^−2^. Another aliquot was also serially diluted, then exposed to γ-rays at different doses of 20, 50, 100, 200, and 500 Gy and then coated on an R2A agar medium. Moreover, the 12C6+ heavy-ion beam generated by HIRFL, Institute of Modern Physics, Chinese Academy of Sciences was used to irradiate the bacterial aliquot with the dose of 500 Gy. The irradiation parameters were as follows: after passing through a 50 μm stainless steel window, 20 μm Mylar film, and 1.3 m air, the 80 MeV/u 12C6+ ions were changed into 76.37 MeV/u; further, the range in water was expected to be 16 mm and the peak position was 15.5 mm. All the above assays had three parallel tests. After 7–14 days of culture, survival colony-forming units (CFUs) were counted and the survival rate (SR) was calculated for the purposes of assessing the ability of ionizing radiation (γ-rays) or non-ionizing (UVC) resistance.

## 3. Results and Discussion

### 3.1. The Phylogenetic Characterization Based on 16S rRNA Gene and UBCG Set

The full-length 16S rRNA gene sequencing and genome data of strain S5-59^T^ were stored in DDBJ/EMBL/GenBank with accession numbers OM809165.1 and CP101740, respectively. The full-length 16S rRNA gene sequencing and genome data of strain S8-45^T^ were stored in DDBJ/EMBL/GenBank with accession numbers MZ314855.1 and CP097253, respectively.

**Figure 1:**
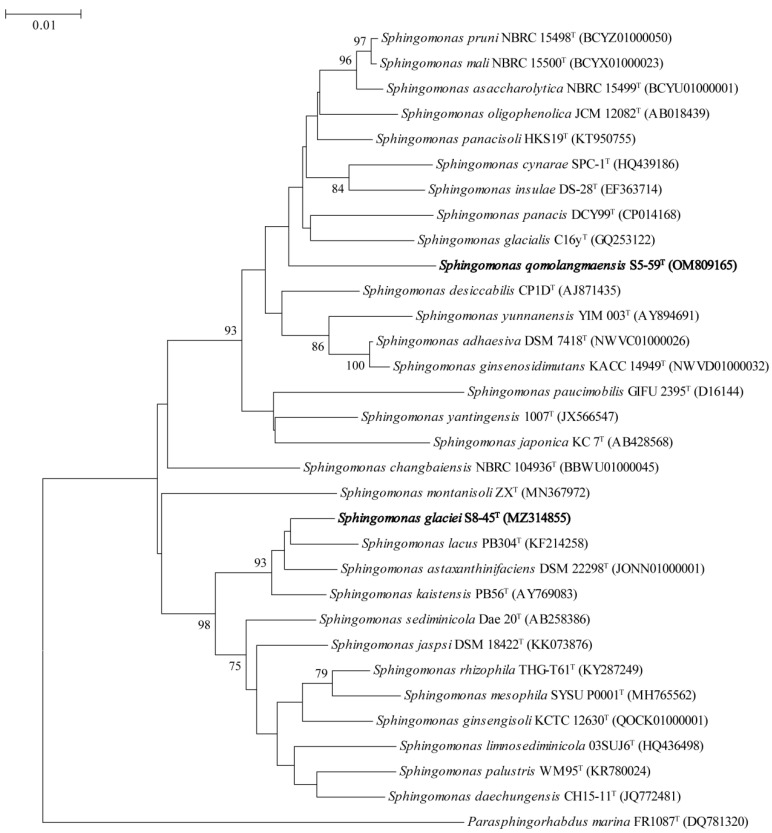
Neighbor-joining phylogenetic tree based on 16S rRNA gene sequences of the strain S5-59^T^, S8-45^T^, and the type strains of other closely related species in the genus *Sphingomonas* and *Parasphingorhabdus*. *Parasphingorhabdus marina* FR1087^T^ (DQ781320) was used as an outgroup. The numbers on the tree indicate the percentages of bootstrap sampling derived from 1000 replications and the bootstrap values higher than 70% are shown. Bar, 0.01 substitutions per nucleotide position.

**Figure 2:**
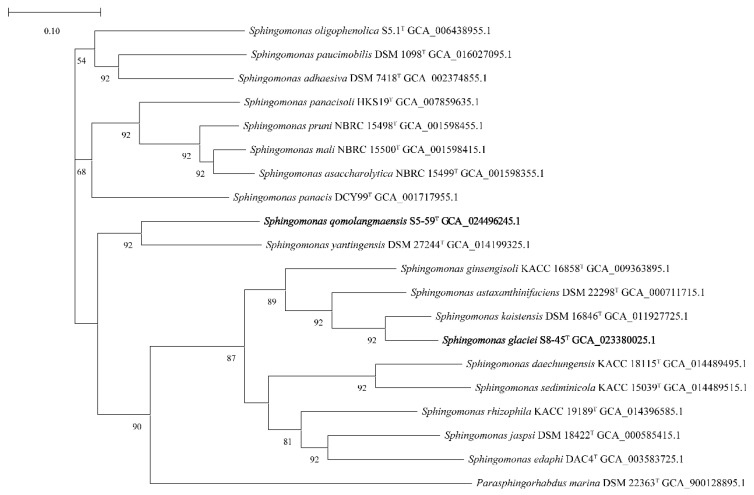
UBCG phylogenetic tree based on the up-to-date core gene set and pipeline of strains S5-59^T^, S8-45^T^, and strains of other closely related species in the genus *Sphingomonas* and *Parasphingorhabdus*. *Parasphingorhabdus marina* FR1087^T^ (DSM 22363^T^) was used as an outgroup. Phylogenetic tree generated with UBCG using the amino acids sequences. The number at the nodes indicates the gene support index (maximum value, 92). The numbers at the nodes indicate the gene support index. Bar, 0.10 substitutions per nucleotide position.

The 16S rRNA gene sequencings of strains S5-59^T^ and S8-45^T^ were compared in the EzTaxon database and affiliated to the phylum *Proteobacteria*. The strains with the highest similarity with strains S5-59^T^ and S8-45^T^ were obtained by comparing the 16S rRNA gene sequencings with the EzTaxon database. Strain S5-59^T^ was 96.83% similar to *S. panacisoli* HKS19^T^, 96.17% similar with *S. asaccharolytica* DSM 10564^T,^, and 96.03% similar with *S. panacis* DCY99^T^. Strain S8-45^T^ was 98.26% similar to *S. kaistensis* PB56^T^, 98.19% similar with *S. astaxanthinifaciens* DSM 22298^T^, and 97.10% similar with *S. ginsengisoli* KCTC 12630^T^. The phylogenetic trees were reconstructed by 4 algorithms with 30 type strains, which were highly related to strains S5-59^T^ and S8-45^T^; further, *Parasphingorhabdus marina* FR1087^T^ was used as an outgroup. The neighbor-joining phylogenetic tree (Figure 1) and the other two trees (Figures S2 and S3), based on 16S rDNA, showed that S8-45^T^, *S. kaistensis* PB56^T^, *S. astaxanthinifaciens* DSM 22298^T^, and *S. lacus* PB304^T^ formed a stable branch, meanwhile strain S5-59^T^ stably formed a cluster. The UBCG phylogenetic tree showed that strain S8-45^T^, *S. kaistensis* DSM16846^T^, *S. astaxanthinifaciens* DSM 22298^T^, and *S. ginsengisoli* KACC 16858^T^ were clustered together. Further, strain S5-59^T^ and *S. yantingensis* DSM 27244^T^ formed a stable branch (Figure 2); this showed that it is different from the three phylogenetic trees based on 16S rRNA. These also suggested that strains S5-59^T^ and S8-45^T^ were the members of the genus *Sphingomonas*.

### 3.2. The Phenotypic Characterization of Strains S5-59^T^ and S8-45^T^

**Figure 3:**
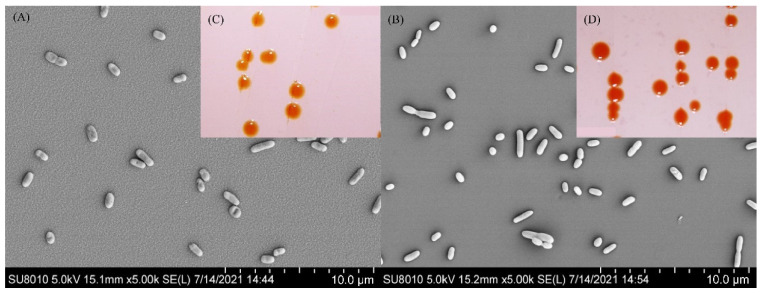
Scanning electron microscope photos showing the morphological structure of the cells of strain S5-59^T^ (**A**), S8-45^T^ (**C**), and plate colonies’ morphology of S5-59^T^ (**B**), and S8-45^T^ (**D**).

The cells of strain S5-59^T^ were aerobic, Gram-negative, non-motile, non-spore-forming, and rod-shaped (0.4–0.6 μm × 0.6–1.3 μm) (Figure 3A). Colonies of strain S5-59^T^ were circular and orange after 72 h of incubation at 30 °C in an R2A agar medium (Figure 3C). The strain S5-59^T^ was capable of growth at temperatures ranging from 10 to 35 °C (optimum 30 °C) and grew well at pH values from 8.0 to 9.0 (optimum 8.0). The strain S5-59^T^ was tolerant to 2% (*w*/*v*) NaCl (optimum 0%) (Table 1). Compared with *S. panacisoli* HKS19^T^, *S. asaccharolytica* DSM 10564^T^, and *S. panacis* DCY99^T^ strain S5-59^T^ had the same range of growth temperature, but a narrower range of growth pH (Table 1). Strain S5-59^T^ was recorded as positive for oxidase activity, but negative for the production of H_2_S and the reduction of nitrate. It could not hydrolyze Tween 20, Tween 80, urea, gelatin, starch, or cellulose. The following carbohydrates were utilized: D-Glucose, D-Lactose, D-Galactose, L-Arabinose, D-Fructose, D-Mannitol, D-Raffinose, D-Rhamnose, and sucrose; further, it must be noted that D-xylose could not be used as the only carbon source. For the utilization of nitrogen sources, strain S5-59^T^ could use L-Aspartate or L-Histidine as the only nitrogen source, but could not use L-Alanine, L-Tyrosine, or L-Cysteine. Strain S5-59^T^ was positive for acid phosphatase, alkaline phosphatase, esterase (C4), esterase lipase (C8), lipase (C14), and naphthol-AS-BI-phosphate, but negative for N-acetyl–β-glucosaminase, α-Mannosidase, and β-Fucosidase (Table 1).

The cells of strain S8-45^T^ were aerobic, Gram-negative, non-motile, non-spore-forming, and rod-shaped (0.3–0.6 μm × 0.6–1.6 μm) (Figure 3B). The colonies of strain S8-45^T^ were round, light-red, and moist after incubation at 30 °C for 72 h (Figure 3D). The growth temperature range of strain S8-45^T^ was 10–35 °C (optimum 30 °C) and could grow in the culture environment with a pH value between 5.0 and 10.0 (optimum 7.0). Additionally, it could grow in the culture environment with a NaCl concentration in the range of 0–2% (*w*/*v*) (optimum 0%) (Table 1). Compared with strain S8-45^T^ and its closest related reference type strains, it possessed a wider growth temperature and pH than them (Table 1). Strain S8-45^T^ was positive for oxidase activity but negative for the production of H_2_S and reduction of nitrate. It could hydrolyze Tween 20, but could not hydrolyze Tween 80, urea, gelatin, starch, or cellulose. Strain S8-45T could use L-Arabinose, D-Fructose, D-Galactose, D-Glucose, D-Lactose, D-Mannitol, D-Raffinose, and D-Rhamnose; further, it could use D-xylose as the only carbon source, and could also weakly use sucrose. For the utilization of nitrogen sources, strain S8-45^T^ could weakly use L-Alanine, and also L-Cysteine as the only nitrogen source. In regard to enzymatic activity, only strain S8-45^T^ was negative for acid phosphatase, which was different from the closely related reference strains. S8-45^T^ was positive for alkaline phosphatase, lipase (C14), and naphthol-AS-BI-phosphate hydrolase, but negative for N-acetyl–β-glucosaminase, α-Mannosidase, and β-Fucosidase (Table 1).

Strain S5-59^T^ had the same range of growth temperature and NaCl tolerance, but a narrower range of growth pH than S8-45^T^ (Table 1). Both of them were positive for oxidase activity but negative for the production of H_2_S, and the reduction of nitrate. L-Alanine and L-Cysteine were utilized by strain S8-45^T^, however not by strain S5-59^T^. Both of them were positive for alkaline phosphatase, esterase (C4), esterase lipase (C8), lipase (C14), and naphthol-AS-BI-phosphate (Table 1).

### 3.3. Chemotaxonomic Characteristics of Strains S5-59^T^ and S8-45^T^

The major cellular fatty acids of strain S5-59^T^ were summed as feature 3 (C_16:1_ *ω*6*c* and/or C_16:1_ *ω*7*c*) and feature 8 (C_18:1_ *ω*7*c*), which were similar to the type strains *S. panacisoli* HKS19^T^, *S. asaccharolytica* DSM 10564^T^, and *S. panacis* DCY99^T^, but the specific content was noted as different (Table S3a). The predominant respiratory menaquinone was ubiquinone-10 (Q-10). Cell wall amino acids of strain S5-59^T^ mainly contained meso-diaminopimelic acid (meso-DAP). The characteristic sugar components of the cell wall were rhamnose, ribose, xylose, and glucose. The polar lipids were diphosphatidylglycerol (DPG), phosphatidylglycerol (PG), phosphatidylethanolamine (PE), an unidentified phospholipid (PL), four unidentified glycolipids (GL), sphingoglycolipid (SGL), and two unidentified lipids (UL) (Figure S3a).

The major fatty acids of strain S8-45^T^ were summed as feature 3 (comprising C_16:1_ *ω*6*c* and/or C_16:1_ *ω*7*c*) and feature 8 (comprising C_18:1_ *ω*6*c* and/or C_18:1_ *ω*7*c*), which were similar to the type strains *S. kaistensis* PB56^T^ and *S. astaxanthinifaciens* DSM 22298^T^, but the specific content was noted as different (Table S3b). The content of unsaturated fatty acids of S8-45^T^, including C_16:1_ *ω*5*c*, C_17:1_ *ω*6*c*, C_17:1_ *ω*8*c*, and C_18:1_ *ω*5*c* was significantly different from the content of other type strains. The predominant respiratory menaquinones of S8-45^T^ were ubiquinone-10 (Q-10), accounting for 96.82%, and respiratory menaquinone ubiquinone-3 (Q-3) accounting for 3.18%. Through the analysis of chemical components of the cell wall, the characteristic DAP component of the cell wall was *meso*-DAP. The characteristic sugar components of the cell wall were ribose, xylose, and galactose. Additionally, the polar lipids of strain S8-45^T^ included DPG, PG, PE, and five ULs (Figure S3b).

### 3.4. The Radiation-Resistant Ability of S5-59^T^ and S8-45^T^

**Figure 4:**
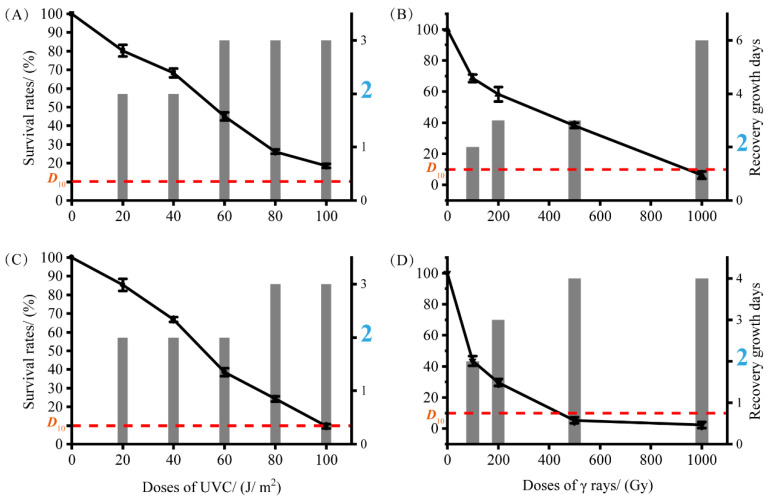
The survival rates of strains S5-59^T^ and S8-45^T^ after irradiation with different doses of UVC and γ rays’ radiation. *D*_10_–values are displayed by the red dash line; the blue numbers on the right *y*-axis of each image represent the normal growth time of the strains without irradiation. (**A**) Survival rates of S5-59^T^ after irradiation by different doses of UV-C; (**B**) survival rates of S5-59^T^ after irradiation by different doses of γ rays; (**C**) survival rates of S8-45^T^ after irradiation by different doses of UV-C; and (**D**) survival rates of S8-45^T^ after irradiation by different doses of γ rays.

To estimate the radiation-resistant ability of the type strains S5-59^T^ and S8-45^T^ after being exposed to UV-NIR (UVC 254 nm) and γ-rays-IR, we chose six reference type strains related to S5-59^T^ and S8-45^T^ as controls (Figure S4). After UVC radiation, type strain S8-45^T^ exhibited a great ability for radiation resistance with 100 J/m^2^ of *D*_10_ value, and S5-59^T^ harbored an even higher radiation resistance (*D*_10_ >100 J/m^2^). Through IR (γ-rays) analysis, Figure 4 shows that S5-59^T^ exhibited stronger radiation resistance (*D*_10_ value close to 100 Gy) than strain S8-45^T^ (*D*_10:_ 400–500 Gy). Both type strains S5-59^T^ and S8-45^T^ harbored a great ability for UVC-NIR resistance, but S5-59^T^ also exhibited stronger γ-rays-IR resistance. To provide a clearer response to the sensitivity of these two strains to radiation, we measured the time taken for bacteria to resume growth after radiation. The time between radiation and the growth of the strain to log phase is also the time for the strain to repair the post-irradiation damage, and perhaps this repair will continue to take more time [66]. Type strain S5-59^T^ was more sensitive to UVC radiation than S8-45^T^, even though both of them harbored a higher radiation resistance to UVC. 

In our study, the growth time of type strain S5-59^T^ when irradiated by UVC doses of more than 60 J/m^2^ was three days (3d), which was more than the normal growth time (2d) without any irradiation (Figure 4). The UVC radiation dose administered to S8-45^T^ that was required in order to cause it to grow in more days than the normal growth rate (2d) was 80 J/m^2^ with 3d. Interestingly, the γ-rays radiation dose administered to S5-59^T^ and S8-45^T^ that was required in order to grow in more days than the normal growth rate was 200 Gy (Figure 4). As such, the higher the irradiated dose, the longer it takes for the bacteria to grow. Figure 4 shows that S5-59^T^ needed six days for restorative growth after 1000 Gy doses of radiation of γ-rays (Figure 4). Generally, whether IR or NIR, cells would repair and grow by themselves after irradiation. The damage caused by IR is somewhat greater than that caused by NIR when a certain irradiation dose was reached [14,67]. Therefore, S5-59^T^ needed more time to repair itself when exposed to larger doses of γ-rays. Strains S5-59^T^ (65.85%) and S8-45^T^ (58.50%) also showed resistance to the 12C6+ heavy-ion beam radiation with a dose of 500 Gy.

### 3.5. The Genome Analysis of Strains S5-59^T^ and S8-45^T^

#### 3.5.1. General Genome Features

**Figure 5:**
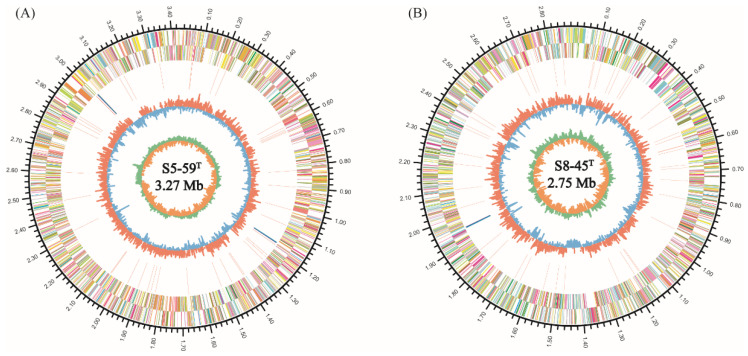
Circular chromosome map and COG functional categories of the strains S5-59^T^ (**A**) and S8-45^T^ (**B**) genomes. The circles show the different descriptions of the content in metabasins, from the outside to inward: the outer circle represents the genome size, the second circle and the third circle represents the predicted protein-coding sequences and CDS regions on the plus and minus strands, respectively. The colors represent COG functional classification. The fourth circle represents the repeated sequence. The fifth circle represents tRNA and rRNA. The sixth circle shows GC content and the seventh circle exhibits the percent of GC-skew. (The detailed clusters of orthologous group information are shown in Table S3).

The complete genome of strain S5-59^T^ contained a single circular chromosome of 3,429,145 bp with a guanine–cytosine (GC) content of 66.42 mol% (Figure 5A). The total number of coding sequencings (CDSs) in strain S5-59^T^ genome was 3142, and the number of RNA was 54, including 48 tRNAs and 2 sets of 5S rRNA, 16S rRNA, and 23S rRNA (Table S2). No plasmid was present in the strain S5-59^T^. The number of CDS in strains S5-59^T^ was less than that of three related strains (Table S2).

The complete genome of strain S8-45^T^ contained a single circular chromosome of 2,880,162 bp with a guanine–cytosine (GC) content of 66.57 mol% (Figure 5B). The total number of CDSs in strain S8-45^T^ genome was 2811, and the number of RNA genes was 50, including 47 tRNAs, one 5S rRNA, one 16S rRNA, and one 23S rRNA. No plasmid was presented in the strain S8-45^T^. The number of CDS in strains S8-45^T^ was also less than that of related reference strains (Table S2).

**Figure 6:**
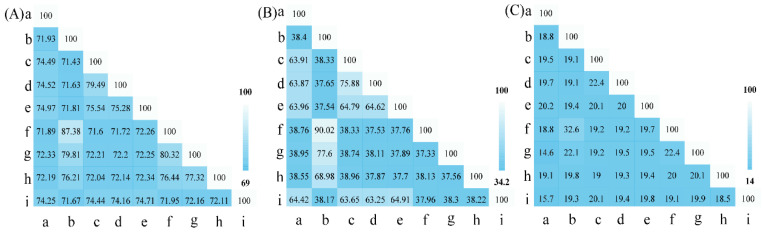
Genome comparisons of strains S5-59^T^, S8-45^T^, and their related reference strains including the OrthoANI value (**A**), AAI value (**B**), and dDDH value (**C**). Further, a–h represent S5-59^T^, S8-45^T^, *Sphingomonas panacisoli* HKS19^T^, *Sphingomonas asaccharolytica* NBRC 15499^T^, *Sphingomonas panacis* DCY99^T^, *Sphingomonas kaistensis* PB56^T^, *Sphingomonas astaxanthinifaciens* DSM 22298^T^, and *Sphingomonas ginsengisoli* KCTC 12630^T^, respectively. Additionally, i was the type species of *Sphingomonas*, i.e., *Sphingomonas paucimobilis* DSM 1098^T^.

ANIb, ANIm, dDDH, and OrthoANI values were calculated to identify the genomic similarities of strains S5-59^T^ and S8-45^T^ to the related reference *Sphingomonas* species, and the type species of the *Sphingomonas* with available genome sequencings (Figure 6 and Figure S5). The sequencing similarity values of the 16S rRNA gene between strains S5-59^T^, S8-45^T^, and their related reference strains were all lower than the species’ division threshold (98.7%) for prokaryotic species [68]. The ANIb values between strains S5-59^T^ and S8-45^T^ were 70.43%; the ANIb values between S5-59^T^ and *S. panacisoli* HKS19^T^ were 73.55%; ANIb between S8-45^T^ and *S. kaistensis* DSM 16846^T^ were 86.86%; the ANIb between the strains S5-59^T^, S8-45^T^, and other *Sphingomonas* species were from 70.32% to 86.86%; the values for ANIm among the strains S5-59^T^, S8-45^T^, and other *Sphingomonas* species were from 88.13% to 84.88%; and the values for Ortho ANI among the strains S5-59^T^, S8-45^T^, and other *Sphingomonas* species were from 71.43% to 87.38%—all of these values were lower than the 95% threshold as defined by prokaryotes [69]. The dDDH values between the strains S5-59^T^, S8-45^T^, and other *Sphingomonas* species were from 14.6% to 32.6%, which were also lower than 70%—the threshold defined by prokaryotic species [70]. These results indicate that strains S5-59^T^ and S8-45^T^ are potentially represented in the novel species of the genus *Sphingomonas*.

#### 3.5.2. General COG Analysis

Clusters of orthologous groups (COGs) are a database including biological orthologous gene clusters (Table S3). The proteins that constitute each orthologous gene cluster are assumed to come from an ancestor protein and have the same function [71]. Therefore, the genome analysis of strain S5-59^T^ illustrated that a total number of 3213 CDSs were distributed into 24 COG functional categories (Figure S6A). The major functional category includes genes that contain cell wall/membrane/envelope biogenesis (COG-M, 221 genes); followed by translation, ribosomal structure, and biogenesis (COG-J, 217 genes); general function prediction only (COG-R, 216 genes); amino acid transport and metabolism (COG-E, 195 genes); signal transduction mechanisms (COG-T, 174 genes); coenzyme transport and metabolism (COG-H, 172 genes); carbohydrate transport and metabolism (COG-G, 168 genes); energy production and conversion (COG-C, 159 genes); lipid transport and metabolism (COG-I, 155 genes); posttranslational modification, protein turnover, and chaperones (COG-O, 153 genes); inorganic ion transport and metabolism (COG-P, 141 genes); replication, recombination, and repair (COG-L, 138 genes); transcription (COG-K, 135 genes); and function unknown (COG-S, 134 genes). The detailed annotation results of COGs containing less than 100 genes are displayed in Figure S6. The genome of strain S8-45^T^ showed that it had 20 COG functional categories including 2813 CDSs, which is 80.86% of all (Figure S6B) categories. Interestingly, one of the major functional categories containing most genes was the unknown function (COG-S, 672 genes) category, which could be indicative of producing various metabolites to respond to the stress of such high-altitude, extremely low temperature, freeze–thaw cycle, and ultraviolet (UV) radiation environment. As such, the specific metabolites produced by the unknown gene clusters need to be further explored. Otherwise, other major functional categories containing genes included amino acid transport and metabolism (COG-E, 174 genes); followed by translation, ribosomal structure, and biogenesis (COG-J, 152 genes); energy production and conversion (COG-C, 139 genes); cell wall/membrane/envelope biogenesis (COG-M 136 genes); signal transduction mechanisms (COG-T, 126 genes); replication, recombination, and repair (COG-L, 111 genes); posttranslational modification, protein turnover, and chaperones (COG-O, 110 genes); and inorganic ion transport and metabolism (COG-P, 108 genes). The remaining annotation results of COGs containing less than 100 genes are displayed in Figure S6.

Comparing the genomes of the strains S5-59^T^ and S8-45^T^, five COG functional categories of strain S5-59^T^, including transcription; lipid transport and metabolism; carbohydrate transport and metabolism; general function prediction only; and coenzyme transport and metabolism all show higher abundance than strain S8-45^T^ when it possesses higher than 100 genes. These proteins that have more genes of functional classification may enhance the greater ionizing radiation resistance (γ-rays) ability and response of S5-59^T^ rather than S8-45^T^ (Figure 4). Moreover, Ye et al. [72] reported that the lipid metabolism of the intracellular structure was extremely sensitive to ionizing radiation as excessive doses of ionizing irradiation could accelerate the cellular lipid peroxidation and cellular lipid generation. A higher abundance of lipid transport and metabolism annotation genes may be an indicator of radiation resistance [73]. Meanwhile, the higher abundant annotation genes of carbohydrate transport and metabolism also elucidated that the more resistant microorganisms were to radiation, then the more carbon metabolically active they were. In addition, they could also utilize diverse carbon sources derived from both microbial cells that have weak resistance to radiation and are killed by the radiation (including C5-C12-containing compounds) [74] in high radiation environments. Nucleotide coenzymes, which were produced from nucleotides (for instance, adenosine, uracil (U), guanine (G), or inosine [75]), could help dot the map of metabolic pathways, providing energy to drive the reactions of the pathway, and especially play an important role in regulating and controlling energy metabolism for DNA repair [76].

#### 3.5.3. Pan-Genome Analysis Related to Radiation Resistance

**Figure 7:**
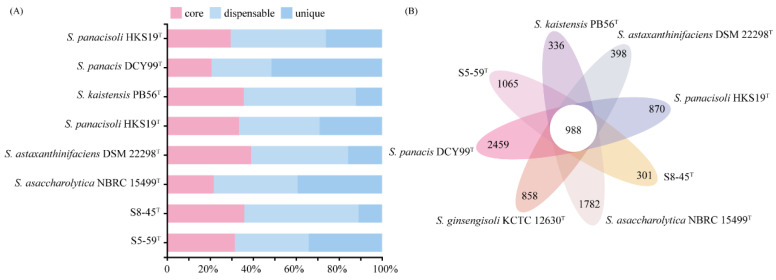
Comparisons of orthologous protein groups in S5-59^T^, S8-45^T^, and six related Sphingomonas genomes. (**A**) Percentage of core, dispensable, and unique genes in each of all eight genomes. (**B**) Venn diagram displaying the number of core and unique genes for each of the S5-59^T^, S8-45^T^, and related type strains.

Pan-genomic studies were carried out to gain an in-depth understanding of the intra-species genomic features of the S5-59^T^, S8-45^T^, and their related species. The Heaps’ law model parameter α estimation was equal to 0.629, which is less than the threshold of 1.00 [77]. Additionally, Figure S7 shows that the gene accumulation curves of the pan genome indicate that the power trend line has not arrived at the platform stage (Figure S7). Therefore, the above results suggest that this genus has an open pangenome. Gene clusters were defined as core (clusters of homologous genes/number of genes present in all samples), dispensable (clusters of homologous genes/number of genes co-occurring in two or more samples), and unique (clusters of homologous genes present in only one sample/number of genes) types (Figure 7A). Comparative analyses based on orthologous groups of proteins revealed that 988 core genes were shared by all 8 *Sphingomonas* genomes (Figure 7B). The percentages of core genes from each *Sphingomonas* species ranged from 20.7% to 39.1%, thereby elucidating a relatively low percentage of common functional proteins in each type species. However, a higher percentage of the dispensable gene ranged from 27.9–53.1% among eight genomes of *Sphingomonas*. In addition, the unique genes had a wide percentage range from 10.9–51.4% (Figure 7A). For the genome of strain S5-59^T^, the unique genes accounted for 34.4% (1065 unique genes), and the S8-45^T^ had just 301 unique genes with a percentage of 10.9% (Figure 7A). The unique genes are usually associated with the species’ genetic diversity, environmental adaptation, and other special characteristics [78].

**Figure 8:**
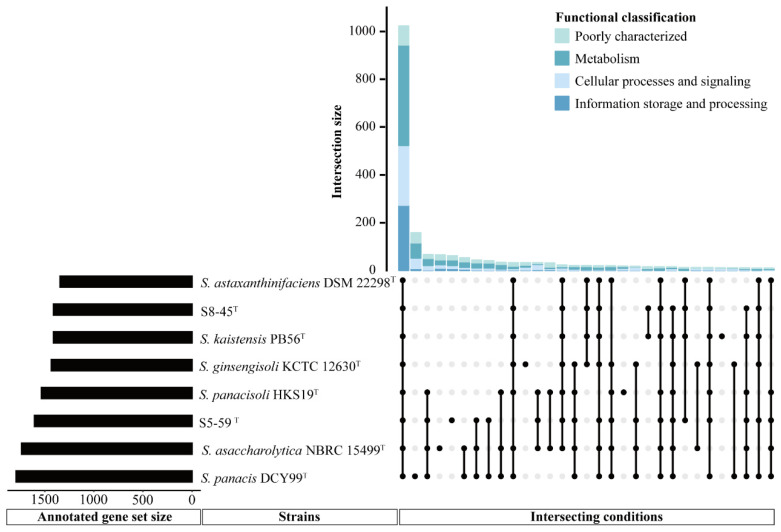
The number and functional gene classification of pan genomes between different *Sphingomonas* strains. The upset plot shows the number and functional classification of the core and unique genes in different *Sphingomonas* strains. The bar chart above represents the number of core and unique genes contained in each type of group. The strip at the bottom left represents the total number of genes in different *Sphingomonas* strains. The dot and line at the bottom right represent the types of different combinations (where only values above 10 and annotated genes are shown; further, unknown genes were not shown).

Generally, orthologous proteins are corresponding genes that evolved from the same ancestral niche in different species, and could also have similar functions. Torre et al. suggested that orthologous protein groups exhibit a similar function of the same gene or pathway to further understand the genetic and evolutionary relationship [79]. To more clearly characterize the similarities and differences between strains S5-59^T^, S8-45^T^, and their related reference species, the number and functional gene classification of pan-genomes between different *Sphingomonas* strains was performed via orthologous comparison analysis of the functional genes (Figure 8). Figure 8 shows that a total of 1023 proteins that corresponded to the annotated genes formed the core genome among all the members of *Sphingomonas* considered here, and each member had its unique genes, except for the undisplayed unknown genes. In the core genome, the gene functional categories were affiliated with metabolism (422 genes), followed by the information storage and processing mechanism (273 genes), the cellular processes and signaling functions (245 genes), and the poorly characterized functions (83 genes). Among these genes—in addition to those related to fatty acid and cell wall biosynthesis; replication; recombination and repair; nucleotide transport; signal transduction; defense mechanisms; and other essential genetic content—genes related to cold shock proteins with *cspA*, *cspB*, and *cshB* were also involved. This, therefore, enabled the *Sphingomonas* strains to obtain the ability of cold resistance [80]. Moreover, the genes encoding heat-shock resistance, for instance *ibpA*, *dank,* and *hrcA*, were annotated in the core gene content, which suggested that the *Sphingomonas* genus could adapt to temperature fluctuations in survival environments during long-term evolution [81]. More importantly, among these core genes, we found that the existence of a large number of genes related to DNA replication, recombination, and repair proteins—including core genes related to DNA replicative proteins such as *dnaB*, *ligA*, *rnhA*, *herA*, *recQ*, *recG*, and *dinG*; core genes related to DNA recombinative proteins, such as *recA*, *recR*, *recO*, *recF*, *radA*, *xerD*, *rarA*, and *rmuC*; and core genes related to DNA repair proteins such as *muts*, *mutL*, *recR*, *recF*, *recN*, *recO*, *radA*, *radC*, *adaA*, and *dinP* [82,83,84,85,86,87,88,89]—can generally enable the genus *Sphingomonas* to defend or repair the radiation-reduced damage such as SSB repair or DSB repair, to some extent. Additionally, the core gene related to the SSB repair protein was also predicted by gene *ssb*, which is an encoded single-stranded DNA-binding protein [90]. In addition, numerous genes encoding oxidative stress resistance proteins such as *yfiH*, *cox11*, *thiO*, *goxB*, *hemN*, and *hslO* were also annotated in the core content, which indicated that the *Sphingomonas* species have a great ROS-scavenging system for defending against oxidative damage caused due to radiation stress [91].

Unlike core genes representing the general features of bacteria, specific genes, such as unique genes, could be used as the main research basis for comparing functional distinctions between different strains [92]. Figure 8 shows that a certain number of specific genes are occupied in S5-59^T^, S8-45^T^, and their related reference strains, of which their specific genes’ counts were 158, 65, 61, 32, 18, and 11 in *S. panacis* DCY99^T^, *S. asaccharolytica* NBRC 15499^T^, S5-59^T^, *S. ginsengisoli* KCTC 12630^T^, *S. panacisoli* HKS19^T^, and *S. kaistensis* PB56^T^, respectively. In addition, only nine specific genes were in S8-45^T^. Some studies have suggested that the differences in the number of these genes may be related to the genome size of the strains, or that the distance of genetic relationships [93] and novel species in extremely harsh environments have more unannotated new genes [94]. Therefore, it is not surprising that there were fewer specific genes in the strains S5-59^T^ and S8-45^T^ than in the other strains. Interestingly, the specific genes in S5-59^T^ were much higher than in S8-45^T^, although they both came from the same extreme environment simply by the difference in altitude. Analyzing the functions of the difference for specific genes in these two strains found that more specific genes in S5-59^T^ were related to metabolism and poorly characterized, which is the same as S8-45^T^. However, genes involved in the DNA replication, recombination, and repair of S5-59^T^ were higher than S8-45^T^; further, more specific genes of S8-45^T^ related to defending oxidation stress (Tables S4 and S5) were found. Additionally, most of the genes related to the function unknown category in S5-59^T^ were associated with antioxidation. Other genes related to the poorly characterized category were associated with adaptation to harsh conditions, such as acid resistance, cold adaptation, pollution degradation, etc. (Table S4). In summary, although the existence of these genes reflected that various specific genes affiliated with strains could enable extremophiles to survive in or adapt to extremely harsh environments, strains that acquired additional stresses from higher altitudes could also promote the unique genetic characteristics of ecological niches, notably radiation resistance [5,11].

#### 3.5.4. Horizontal Gene Transfers Analysis Related to the Ability of Radiation Resistance

Although the strain S5-59^T^ is stronger than S8-45^T^ in the ability of ionizing radiation resistance (γ-rays), both of them have a great ability of non-ionizing radiation resistance (UVC). Cohan and Perry et al. suggest that strains from the same geographical origin are more likely to cause genetic drift [95]. In addition to the core proteins and other orthologous proteins, many non-ortholog proteins were found in both of these two strains. Multiple horizontal gene transfer events occurred in the genomes of S5-59^T^ and S8-45^T^. Nine and six GIs were found in the genomes of S5-59^T^ and S8-45^T^, respectively (Tables S6 and S7). Comparing the gene function analysis of GIs from these two strains, metabolism and membrane transport were the main functions involved in genes. A large number of genes associated with oxidative enzymes and oxidative damage detoxification—for instance Glycine/D-amino acid oxidase, Fe-S oxidoreductase, FAD/FMN-containing lactate dehydrogenase/glycolate oxidase, etc.—were found in both S5-59^T^ and S8-45^T^, which enabled the relief of ROS resulting in oxidative or peroxidative reactions caused by radiation or other stresses [14]. Moreover, we also found that numerous genes associated with DNA repair were also found in GIs of S5-59^T^ and S8-45^T^, for instance: *radC* with UUL81296 and UUR08271; *radC* with UUL82802 and UUR08766; *addA* with UUL84186 and UUR08512; and *ychF* with UUL81408 and UUR09244 (UUL is the prefix of GenBank ID form S5-59^T^ and UUR is the prefix of GenBank ID form S8-45^T^.). Genes affiliated with these GIs showed that S5-59^T^ and S8-45^T^ acquired more genes related to DNA repair, ROS-scavenging, oxidation damage detoxification, etc., in order to adapt, defend, and repair radiation damage. The existence of these genes and gene clusters elucidated that both of them had a consistently homologous genetic evolutionary development in radiation resistance.

### 3.6. Genomic Insights into Two Novel Species Related to Radiation Resistance

Up to now, there have been a small number of members of *Sphingomonas* that were reported to have the ability of radiation resistance. Although, it must be said, the *Sphingomonas* genus does possess multifaceted functions ranging from remediation of environmental contaminations to producing highly beneficial phytohormones, such as sphingan and gellan gum [30]. Asker reported that astaxanthin-producing *Sphingomonas astaxanthinifaciens* TDMA-17^T^ has a radio-tolerant ability [31], and *Sphingomonas radiodurans* S9-5^T^ was reported to be resistant to γ-rays [11]. In our study, strains S5-59^T^ and S8-45^T^, which were isolated from Mount Everest, are characterized by strong irradiation. Thus, the microbes that live there must evolve more adaptation mechanisms for the purposes of better radiation resistance. The irradiation experiments involving S5-59^T^ and S8-45^T^ exhibited this great ability of radiation resistance (Figure 4). After UVC radiation, type strain S8-45^T^ exhibited a great ability of radiation resistance with a 100 J/m^2^ of *D*_10_ value, and S5-59^T^ harbored even higher radiation resistance (*D*_10_ >100 J/m^2^) (Figure 4). In regard to the ionizing radiation of γ-rays, although both of the strains were resistant to γ-rays, type strain S5-59^T^ was more resistant to the IR of γ-rays as shown in Figure 4. Further, the *D*_10_ value (900–1000 Gy) of strain S5-59^T^ is higher than S8-45^T^ (400 -500 Gy). Moreover, strains S5-59^T^ and S8-45^T^ also showed resistance to the 12C^6+^ heavy-ion beam radiation when applied with a dose of 500 Gy. For the purposes of revealing the characterization of radiation-resistant bacteria in high-altitude and strongly irradiated areas, we mined the genomic features of strains S5-59^T^ and S8-45^T^.

Generally, the radiation-resistant bacteria harbored a series of defensive and repair systems to radiation, which is demonstrated in their direct DNA repair systems. In addition, radiation exposure could indirectly produce oxidative damage to cells by increasing protein carbonylation, which could hamper the catalytic activity of proteins and result in cell death, as seen in a H_2_O_2_ treatment dose [14,96]. Therefore, the radiation-resistant bacteria exhibited antioxidant systems, including an efficient enzymatic function consisting of superoxide dismutase (SOD), catalase (CAT), glutathione peroxidase (GPX) etc., and non-enzymatic antioxidant functions consisting of pigments, carotenoids, and deinoxanthin processes for the purposes of scavenging ROS [97,98,99,100]. Among these, DNA repair machineries are considered to be the most important repair system for radiation resistance [14]. Both strains, S5-59^T^ and S8-45^T^, have shown consistently homologous genetic evolutionary development in radiation resistance, which has been determined through horizontal gene transfer analysis. Therefore, multiple and similar copies of DNA repair genes were found in S5-59^T^ and S8-45^T^. For instance, the DNA mismatch repair function is involved in preventing the recombination between the partially homologous DNA sequence genes *mutL* and *mutS* [101]; efficient recombination of donor DNA during transformation genes *radA* and *radC* [102,103]; the proteins involved in suppressing DNA degradation at DSBs encoded by genes *recF* and *recN* [104]; and also the single-stranded DNA-binding protein (SSB), which is involved in not only the protection of single-stranded DNA, but also the recruitment of other proteins for DNA replication, recombination, and repair [105] (Table 2). Moreover, genes *addA* (UUL84186 and UUR08512) and *addB* (UUL83610 and UUR08510) could encode the proteins for DSB repair to increase radiation resistance, which is also essential for the radiation-resistant strain; further, Uvr endonuclease proteins encoded by UUL83357 and UUR09226, could also repair UV radiation-damaged DNA in instances of base excision repair (BER), acting as the same function of DNA glycosylases such as found in *Deincoccus radiodurans* [106,107]. Meanwhile, the SSB protein also expresses a specific function of the DNA repair system, such as in the extended synthesis-dependent strand annealing (ESDSA) process. Further, newly synthesized, long, and single-stranded overhangs generated in the ESDSA process may provide chances to reconstruct a functional genome from chromosomal fragments that became damaged as a result of radiation exposure [108,109]. The occurrence of genes UUL82624, UUL82626, UUR07010, and UUR07012 was predicted to resist radiation involved in the base excision repair (BER) process, according to their functional reports [110]. Interestingly, there were more DNA protection or repair genes annotated in the genome of S5-59^T^, which increased in the process of adapting to the radiation (Table 2).

Remarkably, type strain S5-59^T^ had more distinct genes related to radiation resistance than S8-45^T^ (Table 2), which might be related to the altitude distribution of the ecological niche, as the higher the altitude, the stronger the irradiation. Type strain S8-45^T^ possessed genes UUR07654 and UUR07413 (*uvsE*), which are utilized in order to increase the radiation resistance of bacteria and repair UV-induced DNA damage [111]. In contrast to S8-45^T^, the genome of type strain S5-59^T^ was annotated with more proteins that are involved in DNA protection proteins, except in the case of DNA repair proteins (Table 2). The protein RecX, encoded by gene UUL82877, was involved in suppressing DNA degradation at the DSBs [104,112]; further, the DNA starvation/stationary phase protection protein Dps, encoded by gene UUL82955, could form a DNA-protein crystal that protects DNA from damage by binding DNA in a non-sequence-specific manner [113,114]. In addition, the protein RumC, encoded by *rumC* gene UUL83514, was predicted as either a structural protein that protected DNA against nuclease action or is itself involved in DNA cleavage at the regions of DNA secondary structures [115,116] (Table 2). Moreover, DNA repair proteins RecO (UUL82817) and MmcB (UUL81945) were shown to be involved in the ESDSA process, which is initiated in order to reconstruct a functional genome from chromosomal fragments [105]. Further, as Makharashvili et al. [117] confirmed, the roles of RecO showed two special activities in vitro: DNA annealing and RecA-mediated DNA recombination [117]. In addition, further analysis showed that the strain S5-59^T^, when living in a higher ecological niche, exhibited stronger DNA repair machinery than S8-45^T^, especially in the production of DNA protection proteins when facing the stress of radiation.

Radiation exposure could produce ROS in vivo, which damaged cell survival indirectly using OH•, O_2_^−^, H_2_O_2_, lipid peroxides, etc. [15,16]. Therefore, we suggested that the antioxidant defense systems, including enzymatic activity and non-enzymatic activity antioxidants, could also play a critical role in radiation resistance of type strains S5-59^T^ and S8-45^T^. For enzymatic activity involved in oxidative stress, superoxide dismutase (SOD), catalase (CAT), and glutathione peroxidase (GPX) could be exploited by cells to convert ROS into harmless molecules [99]. Both of these two strains have a great ability to relieve deleterious effects caused by ROS. The number of genes encoding SOD and its family protein catalyzing the conversion of superoxide into oxygen and hydrogen peroxide were equivalent in type strains S5-59^T^ (UUL83639 and UUL83875) and S8-45^T^ (UUR08565 and UUR08996) (Table S8) [118]. The genes UUL83714 and UUL82868 in S5-59^T^ and UUR06761 in S8-45^T^ could encode CAT or CAT/peroxidase HPI, thereby catalyzing the decomposition of hydrogen peroxide to water and oxygen for less oxidative damage [14]. A large number of genes encoding GPX and its relative proteins, were predicted in the genome of S5-59^T^ and S8-45^T^, and the abundance of genes related to GPX in S5-59^T^ was higher than in S8-45^T^ (Table S8). As a selenium-containing antioxidant enzyme, GPX could effectively reduce hydrogen peroxide and lipid peroxides to water and lipid alcohols, respectively [119]. A higher abundance of genes encoding GPX and its relative proteins in type strains S5-59^T^ and S8-45^T^ indicated that GPX plays a significant role in the antioxidation systems that are induced by radiation damage. High GSX content also indicated a possible contribution to chilling tolerance and cold acclimation [120], although these two type strains revealed the presence of multiple specific genes encoding cold-shock proteins, including UUL84132, UUL84136 in S5-59^T,^ and UUR07884, UUR09343, and UUR09349 in S8-45^T^. As a result, we suggest that the GPX could be an indicator of radiation-resistant strains living in high-altitude and extremely cold regions. Further, the major enzymes of ROS-scavenging and other peroxides for oxidative damage, induced by radiation in *Sphingomonas* sp., can also arise from the conditions of such ecological niches.

In addition to the enzymatic activity of proteins involved in radiation-induced oxidative damage, Daly et al. suggest that non-enzymatic antioxidants could also play an efficient ROS-scavenger—for instance, pigments, carotenoids, deinoxanthin, etc. [14,121]. As reported, a major product of the carotenoid synthesis pathway in *D. radiodurans*, deinoxanthin has a higher scavenging ability and protects DNA from oxidative stress [122]. Previous studies indicate that bacteria-lacking genes involved in carotenoid synthesis exhibit increased susceptibility to radiation [14,122]. Asker et al. found that astaxanthin in *Sphingomonas astaxanthinifaciens* plays a critical role in radiation resistance [31], and Liu et al. suggested that the intracellular pigments of *Sphingomonas radiodurance* may diminish the γ-radiation and reduce DNA damage [11]. After analyzing the biosynthetic gene clusters (BGCs) of the secondary metabolites by antiSMASH 6.1.1, our results show that there were genes involved in the carotenoid synthesis, for instance, the C1 gene cluster of S5-59^T^ with zeaxanthin and the C1 gene cluster of S8-45^T^ with carotenoids of antioxidant properties (Table 3). Therefore, we suggest that the predicted carotenoids in type strains S5-59^T^ and S8-45^T^ gene clusters may protect and reduce damage to themselves from oxidation and radiation. Moreover, we also found alkylresorcinol, predicted by BGC C2 in type strain S5-59^T^, has microbial autoregulatory d1 factors for the purposes of enhancing the UV resistance of various DNA molecules [123] (Table 3). Davydova et al. suggest that alkylresorcinols could not only increase the resistance of linearized DNA molecules to UV irradiation, but also enhance the ability to repair DNA damage, thereby preventing both the supercoiled annular–supercoiled relaxed and the supercoiling relaxed–linearized transitions [123]. Therefore, the highly efficient non-enzymatic antioxidant defense system also greatly contributes to the radiation-resistant mechanism of type strains S5-59^T^ and S8-45^T^.

## 4. Conclusions

In this study, we reported two radiation-resistant novel bacteria, *Sphingomonas qomolangmaensis* S5-59^T^ and *Sphingomonas glaciei* S8-45^T^, which were isolated and obtained from the north slope area of Mount Everest. Although several novel radiation-resistant species of *Sphingomonas* have been isolated and reported, there have been few previous studies on the mechanism of radiation-resistance in *Sphingomonas*. Here, we reported the genome insights into two novel *Sphingomonas* species revealing the possible mechanisms of radiation resistance in high-altitude stress areas and glacial environments. It was found that DNA repair machineries are the most important repair system for the radiation resistance trait of S5-59^T^ and S8-45^T^. The genes involved in the DNA repair machineries of S5-59^T^ and S8-45^T^ included the DNA mismatch repair genes *mutL* and *mutS*, the efficient recombination of donor DNA genes *radA* and *radC*, and the single-stranded DNA-binding genes *ssb*, etc. Moreover, type strain S5-59^T^ has more distinct genes related to DNA protection proteins than S8-45^T^, which indicated that the higher ecological niche could activate a greater resistance to radiation in microorganisms. Interestingly, the genes encoding GPX and its relative proteins could also improve the cold adaptation for type strains S5-59^T^ and S8-45^T^. Therefore, we suggest that the coupling of cold adaptation in high-altitude environments and irradiation tolerance may be related to the activity of GPX and its relative proteins in these two strains. In addition, compounds of secondary metabolites such as carotenoids—which include, for example, zeaxanthin and alkylresorcinols—increase the chance of bacterial survival in the environment where higher radiation exists. Therefore, these predicted radio-protective natural products provide possibilities for the development of anti-radiation drugs. In conclusion, genomic analysis and experimental verification indicate that type strains S5-59^T^ and S8-45^T^, in the genus *Sphingomonas*, have the distinct ability to develop radiation resistance. Progress in our understanding of the radiation-resistant mechanism of these bacteria, in this genus, may help in providing important contributions in radiation therapies. Further progress may also effectively set a theoretical basis for the development and potential application of radiation-resistant radio-protective drugs.

### 4.1. Description of Sphingomonas qomolangmaensis sp. nov.

*Sphingomonas qomolangmaensis* sp. nov. (*qomolangma*. en’sis. N.L. masc. adj. *qomolangmaensis* pertaining to Mount Qomolangma, Tibet, China, where the type strain was isolated.)

Cells of type strain S5-59^T^ are aerobic, Gram-negative, non-motile, non-spore-forming, and rod-shaped (0.4–0.6 μm × 0.6–1.3 μm). Colonies of type strain S5-59^T^ are round, and orange after incubation at 30 °C for 72 h in an R2A agar medium. The growth temperature range of type strain S5-59^T^ is 10–35 °C (optimum 30 °C). The growth pH range of type strain S5-59^T^ is 8.0–9.0 (optimum 8.0). Additionally, it can grow with NaCl concentrations of 0–2.0% (*w*/*v*, optimum concentration 0%). Type strain S5-59^T^ exhibited positive oxidase activity and is negative for the production of H_2_S, reduction of nitrate, and hydrolysis of urea, Tween 20, Tween 80, gelatin, starch, and cellulose. Type strain S5-59^T^ can use L-Arabinose, D-Fructose, D-Glucose, D-Galactose, D-Lactose, D-Mannitol, D-Raffinose, D-Rhamnose, and sucrose as carbon sources, but cannot use D-xylose. For utilization as nitrogen sources, type strain S5-59^T^ can use L-Aspartate, L-Histidine, but cannot use L-Alanine, L-Tyrosine, and L-Cysteine. For enzymatic activities, type strain S5-59^T^ is positive for alkaline phosphatase, acid phosphatase, esterase (C4), naphthol-AS-BI-phosphohydrolase, lip esterase (C8), and lipase (C14); further, it is negative for α-Mannosidase, β-Fucosidase, and N-acetyl-β-glucosaminase.

The major cellular fatty acids of type strain S5-59^T^ are summed as feature 3 (comprising C_16:1_ *ω*6*c* and/or C_16:1_ *ω*7*c*), and feature 8 (C_18:1_ *ω*7*c*). Ubiquinone-10 (Q-10) is the predominant respiratory menaquinone. The characteristic sugar components of the cell wall are rhamnose, ribose, xylose, and glucose. The polar lipids of type strain S5-59^T^ are diphosphatidylglycerol (DPG), phosphatidylglycerol (PG), phosphatidylethanolamine (PE), unidentified phospholipid (PL), four unidentified glycolipids (GL), sphingoglycolipid (SGL), and two unidentified lipids (UL).

The type strain, S5-59^T^ (=JCM 35564^T^ =GDMCC 1.3193^T^), was isolated from moraine from the north slope area of Mount Everest (28.02° N, 86.56° E), PR China. The DNA G+C content of the type strain is 66.4%. The full-length 16S rRNA gene sequencing and genome data of strain S5-59^T^ were stored in DDBJ/EMBL/GenBank with accession numbers OM809165.1 and CP101740, respectively.

### 4.2. Description of Sphingomonas glaciei sp. nov.

*Sphingomonas glaciei* sp. nov. (gla.ci. e’i. L. gen. n. *glaciei* of ice, referring to the frozen environment from which the type strain was isolated).

Cells of type strain S8-45^T^ aerobic, Gram-negative, non-motile, non-spore-forming, and rod-shaped (0.3–0.6 μm × 0.6–1.6 μm). The colonies of type strain S8-45^T^ are round, light-red, and moist after incubation at 30 °C for 72 h on R2A agar medium. Growth occurs at 10–35 °C (optimum temperature 30 °C), at pH 5.0–10.0 (optimum pH 7.0), and at NaCl concentrations of 0–2.0% (*w*/*v*, optimum concentration 0%). The carbon sources that can be used by type strain S8-45^T^ are L-Arabinose, D-Fructose, D-Galactose, D-Glucose, D-Lactose, D-Mannitol, D-Raffinose, D-Rhamnose, and D-xylose, and it can also weakly use sucrose. Type strain S8-45^T^ can weakly use L-Alanine, and L-Cysteine as the only nitrogen source. Type strain S8-45^T^ is positive for oxidase activity and Tween 20, but negative for the production of H_2_S, reduction of nitrate, hydrolysis of urea, Tween 80, gelatin, starch, and cellulose. Type strain S8-45^T^ is negative for acid phosphatase, N-acetyl–β-glucosaminase, α-Mannosidase, and β-Fucosidase, but positive for alkaline phosphatase, esterase (C4), lip esterase (C8), and Naphthol-AS-BI-phosphate hydrolase.

The major fatty acids of type strain S8-45^T^ are summed as feature 3 (comprising C_16:1_ *ω*6*c* and/or C_16:1_ *ω*7*c*) and feature 8 (comprising C_18:1_ *ω*6*c* and/or C_18:1_ *ω*7*c*). The predominant respiratory menaquinone of S8-45^T^ is ubiquinone-10 (Q-10). The characteristic sugar components of the cell wall are ribose, xylose, and galactose. The polar lipids of strain S8-45^T^, include diphosphatidyl glycerol (DPG); phosphatidyl glycerol (PG); phosphatidyl ethanolamine (PE); and five unidentified lipids (UL).

The type strain, S8-45^T^ (=JCM 34749^T^ =GDMCC 1.2715^T^) was isolated from moraine from the north slope area of Mount Everest (28.02° N, 86.56° E), PR China. The DNA G+C content of the type strain is 66.6%. The full-length 16S rRNA gene sequencing and genome data of strain S8-45^T^ were stored in DDBJ/EMBL/GenBank with accession numbers MZ314855.1 and CP097253, respectively.

## Figures and Tables

**Table 1:** Phenotypic characteristics of strain S5-59^T^, strain S8-45^T^, and their closely related type strains in the genus *Sphingomonas*.

Characteristics	1	2	3	4	5	6	7	8
Isolation source	Moraine	Soil	Plants	Rusty ginseng	Moraine	Soil	Misasa	Soil
Colony color	Orange	Yellow	Light yellow	Light yellow	Light red	Pink-red	Deep red	Deep orange
Growth temperature (°C) Range (optimum)	10–35 (30)	10–35 (30)	10–35 (30)	10–35 (30)	10–35 (30)	20–35 (30)	40–45 (28)	15–35 (30)
pH range (optimum)	8–9 (8)	6–9 (7)	5–10 (8)	5–7 (6)	5–10 (7)	5–8 (7)	6–8 (7)	6–7 (7)
NaCl tolerance range (%, *w*/*v*) (optimum)	0–2 (0)	0–1 (0)	0–2 (0)	0–1 (0)	0–2 (0)	0–4 (1)	0–3 (1)	0–1 (0)
Oxidase activity	+	+	+	+	+	+	−	−
Hydrolysis of:								
Tween 20	−	−	−	−	+	−	+	−
Gelatin	−	−	−	−	−	−	+	+
Starch	−	−	−	−	−	−	+	−
Utilization as carbon sources								
L-Arabinose	+	+	W	+	+	−	+	−
D-Fructose	+	−	−	+	+	+	−	−
D-Glucose	W	−	−	+	+	+	−	+
D-Lactose	W	−	−	+	+	W	−	−
D-Galactose	+	−	−	+	+	W	−	−
D-Mannitol	+	+	−	−	+	−	−	−
D-Raffinose	+	−	+	−	+	−	−	−
D-Rhamnose	+	−	−	+	+	−	−	−
Sucrose	+	+	−	+	W	−	−	−
D-xylose	−	−	+	−	+	−	−	−
Utilization as nitrogen sources								
L-Alanine	−	−	+	−	W	−	+	−
L-Aspartate	+	−	+	+	−	W	−	+
L-Histidine	+	+	+	+	−	−	−	+
L-Tyrosine	−	−	+	−	−	−	W	+
L-Cysteine	−	−	+	−	W	−	+	−
Enzymatic activity								
Alkaline phosphatase	+	−	+	+	+	−	+	−
Esterase (C4)	+	−	+	W	+	+	+	+
Acid phosphatase	+	−	+	+	−	+	W	+
N-acetyl-β-glucosaminase	−	−	+	−	−	−	+	−
naphtholAS-BI-phosphohydrolase	+	−	+	+	+	+	+	+
Motility	−	−	+	+	−	−	+	−

Strains: 1. S5-59^T^; 2. *Sphingomonas panacisoli* HKS19^T^; 3. *S. asaccharolytica* DSM 10564^T^; 4. *S. panacis* DCY99^T^; 5. S8-45^T^; 6. *S. kaistensis* PB56^T^; 7. *S. astaxanthinifaciens* DSM 22298^T^; and 8. *S. ginsengisoli* KCTC 12630^T^. All related type strains of S5-59^T^ were positive for oxidase activity, lip esterase (C8), lipase (C14), and negative in production of H_2_S, reduction of nitrate, urea, tween 80, cellulose, α-Mannosidase, β-Fucosidase, and Sporemation. All related type strains of S8-45^T^ were positive for lip esterase (C8), lipase (C14), and negative in production of H_2_S, reduction in nitrate, urea, tween 80, cellulose, α-Mannosidase, β-Fucosidase, and Sporemation. All data represented were obtained from this study. +, Positive; −, negative; W, weakly positive.

**Table 2:** List of genes encoding radiation-resistant DNA repair response proteins in the genomes of type strains S5-59^T^ and S8-45^T^.

GenBank ID of S5-59^T^	Gene Symbol	Description	GenBank ID of S8-45^T^	Gene Symbol	Description
UUL83393	*mutL*	DNA mismatch repair endonuclease MutL	UUR08469	*mutL*	DNA mismatch repair endonuclease MutL
UUL84211	*mutS*	DNA mismatch repair protein MutS	UUR07385	*mutS*	DNA mismatch repair protein MutS
UUL81296	*radA*	DNA repair protein RadA	UUR08271	*radA*	DNA repair protein RadA
UUL82802	*radC*	DNA repair protein RadC	UUR08766	*radC*	DNA repair protein RadC
UUL81325	*recN*	DNA repair protein RecN	UUR06914	*recN*	DNA repair protein RecN
UUL82320	*recF*	DNA replication/repair protein RecF	UUR09455	*recF*	DNA replication/repair protein RecF
UUL84186	*addA*	Double-strand break repair helicase AddA	UUR08512	*addA*	Double-strand break repair helicase AddA
UUL83610	*addB*	Double-strand break repair protein AddB	UUR08510	*addB*	Double-strand break repair protein AddB
UUL82209	*ssb*	Single-stranded DNA-binding protein	UUR07233	*ssb*	Single-stranded DNA-binding protein
UUL82624	-	Ligase-associated DNA damage response exonuclease	UUR07010	-	Ligase-associated DNA damage response exonuclease
UUL82626	-	Ligase-associated DNA damage response DEXH box helicase	UUR07012	-	Ligase-associated DNA damage response DEXH box helicase
UUL81513	-	DNA repair protein	UUR07773	-	DNA repair protein
UUL84288	*dinB*	DNA polymerase IV	UUR07654	-	putative DNA modification/repair radical SAM protein
UUL83514	*rumC*	DNA recombination protein RmuC	UUR07413	*uvsE*	UV DNA damage repair endonuclease UvsE
UUL82817	*recO*	DNA repair protein RecO	UUR09226	-	UvrD-helicase domain-containing protein
UUL82877	-	RecX family transcriptional regulator			
UUL81945	-	MmcB family DNA repair protein			
UUL82955	-	DNA starvation/stationary phase protection protein Dps			
UUL83357	-	UvrB/UvrC motif-containing protein			

**Table 3:** The predicted biosynthetic gene clusters related to radiation resistance and the antioxidant function of type strains S5-59^T^ and S8-45^T^.

Type Strain	BGCs Predicted by antiSMASH	Type	Predicted Compounds	Predicted Function
S5-59^T^	C1	Terpene	Zeaxanthin	Antioxidant
	C5	Saccharide	lipopolysaccharide	Antioxidant
S8-45^T^	C1	Terpene	Carotenoid	Antioxidant
	C2	Polyketide	Alkylresorcinol	UV-resistance
Predicted structures	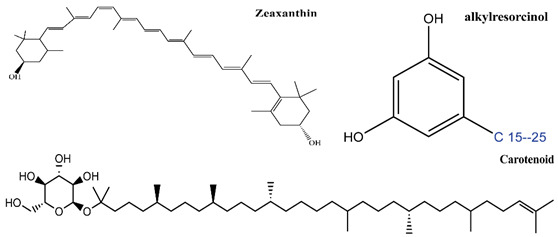			

## Data Availability

The GenBank/EMBL/DDBJ accession numbers for the genome and 16S rRNA gene sequences of strain S5-59^T^ are CP101740 and OM809165.1, respectively. The GenBank/EMBL/DDBJ accession numbers for the genome and 16S rRNA gene sequences of strain S8-45^T^ are CP097253 and MZ314855.1, respectively.

## Supplementary Materials

The following supporting information can be downloaded at: https://www.mdpi.com/article/10.3390/microorganisms10102037/s1. Figure S1: Minimum-evolution phylogenetic tree based on 16S rRNA gene sequences of the strain S5-59^T^, S8-45^T^, and the type strains of other closely related species in the genus *Sphingomonas* and *Parasphingorhabdus*. *Parasphingorhabdus marina* FR1087^T^ (DQ781320) was used as an outgroup. The numbers on the tree indicate the percentages of bootstrap sampling derived from 1000 replications and the bootstrap values higher than 70% are shown. Bar, 0.01 substitutions per nucleotide position. Figure S2: Maximum-likelihood phylogenetic tree based on 16S rRNA gene sequences of the strain S5-59^T^, S8-45^T^, and the type strains of other closely related species in the genus *Sphingomonas* and *Parasphingorhabdus*. *Parasphingorhabdus marina* FR1087^T^ (DQ781320) was used as an outgroup. The numbers on the tree indicate the percentages of bootstrap sampling derived from 1000 replications and the bootstrap values higher than 70% are shown. Bar, 0.01 substitutions per nucleotide position. Figure S3a: Polar lipid profile of strain S5-59^T^. Total lipids were visualized after two-dimensional TLC and applying 5% ethanolic molybdatophosphoric acid. The solvent system was phosphomolybdic acid (A), molybdenum blue (B), indigohydrone (C), and α-naphthol (D) from left to right and top to bottom. Abbreviations: DPG, diphosphatidylglycerol; PG, phosphatidylglycerol; PE, phosphatidylethanolamine; PL1, unidentified phospholipid; GL1-4, unidentified glycolipids; SGL, sphingoglycolipid; L1-2, unidentified lipid. Figure S3b: Polar lipid profile of strain S8-45^T^. Total lipids were visualized after two-dimensional TLC and applying 5% ethanolic molybdatophosphoric acid. The solvent system was phosphomolybdic acid (A), molybdenum blue (B), indigohydrone (C), and α-naphthol (D) from left to right and top to bottom. Abbreviations: DPG, diphosphatidyl glycerol; PG, phosphatidyl glycerol; PE, phosphatidyl ethanolamine; UL 1-5, unidentified lipids. Figure S4: The survival rates of strains S5-59^T^, S8-45^T^, and reference strains after irradiation with different dose of UVC (A) and γ rays’ (B) radiation. D10 values were displayed by red dash line. (A) S5-59^T^; (B) *S. panacisoli* HKS19^T^; (C) *S. asaccharolytica* NBRC 15499^T^; (D) *S. panacis* DCY99^T^; (E) S8-45^T^; (F) *S. kaistensis* PB56^T^; (G) *S. astaxanthinifaciens* DSM 22298^T^, and (H) *S. ginsengisoli* KCTC 12630^T^. Figure S5: Genome comparisons of strains S5-59^T^, S8-45^T^, and their related reference strains including ANIb value (A) and ANIm value (B). Further, a-h represents S5-59^T^, S8-45^T^, *S. panacisoli* HKS19^T^, *S. asaccharolytica* NBRC 15499^T^, *S. panacis* DCY99^T^, *S. kaistensis* PB56^T^, *S. astaxanthinifaciens* DSM 22298^T^, and *S. ginsengisoli* KCTC 12630^T^, respectively. In addition, i is the type species of *Sphingomonas*, *S. paucimobilis* DSM 1098^T^. Figure S6: COG functional categories of the strains S5-59^T^ (A) and S8-45^T^ (B) genomes. The description of the COG type is shown in Table S3. Figure S7: Characteristic curves of the pan genome and core genome of S5-59^T^ (A) and S8-45^T^ (B). Table S1: Whole cellular fatty acids composition of S5-59^T^ and the closely related type strains of the genus *Sphingomonas*. Table S2: General genomic characteristics comparison of strains S5-59^T^ and S8-45^T^ with closely related type strains. Table S3: The description of the COG type. Table S4: Partial unique genes of pan genome in strain S5-59^T^. Table S5: The specific genes of pan-genome in strain S8-45^T^. Table S6: Features of the GIs found in the genome of *S. qomolangmaensis* S5-59^T^. Table S7: Features of the GIs found in the genome of *S. glaciei* S8-45^T^. Table S8: List of partial genes encoding enzymatic activity antioxidant proteins involved in radiation-induced oxidative stress in the genomes of type strains S5-59^T^ and S8-45^T^.

## References

[1] Lieberman P., Morey A., Hochstadt J., Larson M., Mather S. (2005). Mount Everest: A space analogue for speech monitoring of cognitive deficits and stress. Aviat. Space Environ. Med..

[2] Moore G.W., Semple J.L. (2009). The impact of global warming on Mount Everest. High Alt. Med. Biol..

[3] Sood U., Dhingra G.G., Anand S., Hira P., Kumar R., Kaur J., Verma M., Singhvi N., Lal S., Rawat C.D. (2022). Microbial Journey: Mount Everest to Mars. Indian J. Microbiol..

[4] Ji M., Kong W., Jia H., Delgado-Baquerizo M., Zhou T., Liu X., Ferrari B.C., Malard L., Liang C., Xue K. (2022). Polar soils exhibit distinct patterns in microbial diversity and dominant phylotypes. Soil Biol. Biochem..

[5] Yang R., Zhang B., Xu Y., Zhang G., Liu Y., Zhang D., Zhang W., Chen T., Liu G. (2022). Genomic insights revealed the environmental adaptability of *Planococcus halotolerans* Y50 isolated from petroleum-contaminated soil on the Qinghai-Tibet Plateau. Gene.

[6] Huang J., Huang Y. (2021). *Lentzea tibetensis* sp. nov., a novel Actinobacterium with antimicrobial activity isolated from soil of the Qinghai-Tibet Plateau. Int. J. Syst. Evol. Microbiol..

[7] Dong K., Yang J., Lu S., Pu J., Lai X.H., Jin D., Li J., Zhang G., Wang X., Zhang S. (2020). *Microbacterium wangchenii* sp. nov., isolated from faeces of Tibetan gazelles (*Procapra picticaudata*) on the Qinghai-Tibet Plateau. Int. J. Syst. Evol. Microbiol..

[8] Zhang B., Tang S., Yang R., Chen X., Zhang D., Zhang W., Li S., Chen T., Liu G., Dyson P. (2019). *Streptomyces dangxiongensis* sp. nov., isolated from soil of Qinghai-Tibet Plateau. Int. J. Syst. Evol. Microbiol..

[9] Zhang G., Niu F., Busse H.J., Ma X., Liu W., Dong M., Feng H., An L., Cheng G. (2008). *Hymenobacter psychrotolerans* sp. nov., isolated from the Qinghai--Tibet Plateau permafrost region. Int. J. Syst. Evol. Microbiol..

[10] Kang S., Zhang Q., Zhang Y., Guo W., Ji Z., Shen M., Wang S., Wang X., Tripathee L., Liu Y. (2022). Warming and thawing in the Mt. Everest region: A review of climate and environmental changes. Earth-Sci. Rev..

[11] Liu Y., Chen T., Cui X., Xu Y., Hu S., Zhao Y., Zhang W., Liu G., Zhang G. (2022). *Sphingomonas radiodurans* sp. nov., a novel radiation-resistant bacterium isolated from the north slope of Mount Everest. Int. J. Syst. Evol. Microbiol..

[12] Zhang B., Yang R., Zhang G., Liu Y., Zhang D., Zhang W., Chen T., Liu G. (2020). Characteristics of *Planococcus antioxidans* sp. nov., an antioxidant-producing strain isolated from the desert soil in the Qinghai-Tibetan Plateau. MicrobiologyOpen.

[13] Halliwell B., Gutteridge J.M.C. (2015). Free Radicals in Biology and Medicine.

[14] Jung K.W., Lim S., Bahn Y.S. (2017). Microbial radiation-resistance mechanisms. J. Microbiol..

[15] Close D.M., Nelson W.H., Bernhard W.A. (2013). DNA damage by the direct effect of ionizing radiation: Products produced by two sequential one-electron oxidations. J. Phys. Chem. A.

[16] Madian A.G., Regnier F.E. (2010). Proteomic identification of carbonylated proteins and their oxidation sites. J. Proteome Res..

[17] Azzam E.I., Jay-Gerin J.P., Pain D. (2012). Ionizing radiation-induced metabolic oxidative stress and prolonged cell injury. Cancer Lett..

[18] Bruskov V.I., Karp O.E., Garmash S.A., Shtarkman I.N., Chernikov A.V., Gudkov S.V. (2012). Prolongation of oxidative stress by long-lived reactive protein species induced by X-ray radiation and their genotoxic action. Free Radic. Res..

[19] Ranawat P., Rawat S. (2017). Radiation resistance in thermophiles: Mechanisms and applications. World J. Microbiol. Biotechnol..

[20] Yuan M., Zhang W., Dai S., Wu J., Wang Y., Tao T., Chen M., Lin M. (2009). *Deinococcus gobiensis* sp. nov., an extremely radiation-resistant bacterium. Int. J. Syst. Evol. Microbiol..

[21] Tsai C.H., Liao R., Chou B., Contreras L.M. (2015). Transcriptional analysis of *Deinococcus radiodurans* reveals novel small RNAs that are differentially expressed under ionizing radiation. Appl. Environ. Microbiol..

[22] Jeon S.H., Kang M.S., Joo E.S., Kim E.B., Lim S., Jeong S.W., Jung H.Y., Srinivasan S., Kim M.K. (2016). *Deinococcus persicinus* sp. nov., a radiation-resistant bacterium from soil. Int. J. Syst. Evol. Microbiol..

[23] Daly M.J., Minton K.W. (1995). Resistance to radiation. Science.

[24] Matsumura Y., Ananthaswamy H.N. (2004). Toxic effects of ultraviolet radiation on the skin. Toxicol. Appl. Pharmacol..

[25] Liu Y., Chen T., Li J., Wu M., Liu G., Zhang W., Zhang B., Zhang S., Zhang G. (2022). High Proportions of Radiation-Resistant Strains in Culturable Bacteria from the Taklimakan Desert. Biology.

[26] Yabuuchi E., Yano I., Oyaizu H., Hashimoto Y., Ezaki T., Yamamoto H. (1990). Proposals of *Sphingomonas paucimobilis* gen. nov. and comb. nov., *Sphingomonas parapaucimobilis* sp. nov., *Sphingomonas yanoikuyae* sp. nov., *Sphingomonas adhaesiva* sp. nov., *Sphingomonas capsulata* comb. nov., and two genospecies of the genus *Sphingomonas*. Microbiol. Immunol..

[27] Fan Q.M., Zhang R.G., Chen H.Y., Feng Q.Q., Lv J. (2019). Sphingomonas floccifaciens sp. nov., isolated from subterranean sediment. Int. J. Syst. Evol. Microbiol..

[28] Dong L., Li S., Lian W.H., Wei Q.C., Mohamad O.A.A., Hozzein W.N., Ahmed I., Li W.J. (2022). *Sphingomonas arenae* sp. nov., isolated from desert soil. Int. J. Syst. Evol. Microbiol..

[29] Zhang D.C., Busse H.J., Liu H.C., Zhou Y.G., Schinner F., Margesin R. (2011). *Sphingomonas glacialis* sp. nov., a psychrophilic bacterium isolated from alpine glacier cryoconite. Int. J. Syst. Evol. Microbiol..

[30] Asaf S., Numan M., Khan A.L., Al-Harrasi A. (2020). Sphingomonas: From diversity and genomics to functional role in environmental remediation and plant growth. Crit. Rev. Biotechnol..

[31] Asker D., Awad T.S., Beppu T., Ueda K. (2018). Purification and Identification of Astaxanthin and Its Novel Derivative Produced by Radio-tolerant Sphingomonas astaxanthinifaciens. Methods Mol. Biol..

[32] Reasoner D.J., Geldreich E.E. (1985). A new medium for the enumeration and subculture of bacteria from potable water. Appl. Environ. Microbiol..

[33] Shirling E.B., Gottlieb D. (1966). Methods for characterization of Streptomyces species1. Microbiol. Soc..

[34] Williams S.T., Goodfellow M., Alderson G., Wellington E.M., Sneath P.H., Sackin M.J. (1983). Numerical classification of Streptomyces and related genera. J. Gen. Microbiol..

[35] Kurup P.V., Schmitt J.A. (1973). Numerical taxonomy of Nocardia. Can. J. Microbiol..

[36] Collins M.D., Pirouz T., Goodfellow M., Minnikin D.E. (1977). Distribution of menaquinones in actinomycetes and corynebacteria. J. Gen. Microbiol..

[37] Lechevalier H.A., Lechevalier M.P., Gerber N.N., Perlman D. (1971). Chemical Composition as a Criterion in the Classification of Actinomycetes. Advances in Applied Microbiology.

[38] Staneck J.L., Roberts G.D. (1974). Simplified approach to identification of aerobic actinomycetes by thin-layer chromatography. Appl. Microbiol..

[39] Minnikin D.E., O’Donnell A.G., Goodfellow M., Alderson G., Athalye M., Schaal A., Parlett J.H. (1984). An integrated procedure for the extraction of bacterial isoprenoid quinones and polar lipids. J. Microbiol. Methods.

[40] Sasser M. (1990). MIDI technical note 101. Identification of Bacteria by Gas Chromatography of Cellular Fatty Acids.

[41] Weisburg W.G., Barns S.M., Pelletier D.A., Lane D.J. (1991). 16S ribosomal DNA amplification for phylogenetic study. J. Bacteriol..

[42] Larkin M.A., Blackshields G., Brown N.P., Chenna R., McGettigan P.A., McWilliam H., Valentin F., Wallace I.M., Wilm A., Lopez R. (2007). Clustal W and Clustal X version 2.0. Bioinformatics.

[43] Tamura K., Stecher G., Kumar S. (2021). MEGA11: Molecular Evolutionary Genetics Analysis Version 11. Mol. Biol. Evol..

[44] Saitou N., Nei M. (1987). The neighbor-joining method: A new method for reconstructing phylogenetic trees. Mol. Biol. Evol..

[45] Felsenstein J. (1981). Evolutionary trees from DNA sequences: A maximum likelihood approach. J. Mol. Evol..

[46] Nishimaki T., Sato K. (2019). An Extension of the Kimura Two-Parameter Model to the Natural Evolutionary Process. J. Mol. Evol..

[47] Na S.I., Kim Y.O., Yoon S.H., Ha S.M., Baek I., Chun J. (2018). UBCG: Up-to-date bacterial core gene set and pipeline for phylogenomic tree reconstruction. J. Microbiol..

[48] Luo R., Liu B., Xie Y., Li Z., Huang W., Yuan J., He G., Chen Y., Pan Q., Liu Y. (2012). SOAPdenovo2: An empirically improved memory-efficient short-read de novo assembler. GigaScience.

[49] Wick R.R., Judd L.M., Gorrie C.L., Holt K.E. (2017). Unicycler: Resolving bacterial genome assemblies from short and long sequencing reads. PLoS Comput. Biol..

[50] Lee I., Ouk Kim Y., Park S.C., Chun J. (2016). OrthoANI: An improved algorithm and software for calculating average nucleotide identity. Int. J. Syst. Evol. Microbiol..

[51] Richter M., Rosselló-Móra R., Oliver Glöckner F., Peplies J. (2016). JSpeciesWS: A web server for prokaryotic species circumscription based on pairwise genome comparison. Bioinformatics.

[52] Chun J., Oren A., Ventosa A., Christensen H., Arahal D.R., da Costa M.S., Rooney A.P., Yi H., Xu X.W., De Meyer S. (2018). Proposed minimal standards for the use of genome data for the taxonomy of prokaryotes. Int. J. Syst. Evol. Microbiol..

[53] Yoon S.H., Ha S.M., Kwon S., Lim J., Kim Y., Seo H., Chun J. (2017). Introducing EzBioCloud: A taxonomically united database of 16S rRNA gene sequences and whole-genome assemblies. Int. J. Syst. Evol. Microbiol..

[54] Rodriguez-R L.M., Konstantinidis K.T.J.M.M. (2014). Bypassing Cultivation to Identify Bacterial Species: Culture-independent genomic approaches identify credibly distinct clusters, avoid cultivation bias, and provide true insights into microbial species. Microbe.

[55] Meier-Kolthoff J.P., Auch A.F., Klenk H.P., Göker M. (2013). Genome sequence-based species delimitation with confidence intervals and improved distance functions. BMC Bioinform..

[56] Tatusova T., DiCuccio M., Badretdin A., Chetvernin V., Nawrocki E.P., Zaslavsky L., Lomsadze A., Pruitt K.D., Borodovsky M., Ostell J. (2016). NCBI prokaryotic genome annotation pipeline. Nucleic Acids Res..

[57] Mi Z., Zhongqiang C., Caiyun J., Yanan L., Jianhua W., Liang L. (2022). Circular RNA detection methods: A minireview. Talanta.

[58] Overbeek R., Olson R., Pusch G.D., Olsen G.J., Davis J.J., Disz T., Edwards R.A., Gerdes S., Parrello B., Shukla M. (2014). The SEED and the Rapid Annotation of microbial genomes using Subsystems Technology (RAST). Nucleic Acids Res..

[59] Kanehisa M., Goto S., Kawashima S., Okuno Y., Hattori M. (2004). The KEGG resource for deciphering the genome. Nucleic Acids Res..

[60] Cantalapiedra C.P., Hernández-Plaza A., Letunic I., Bork P., Huerta-Cepas J. (2021). eggNOG-mapper v2: Functional Annotation, Orthology Assignments, and Domain Prediction at the Metagenomic Scale. Mol. Biol. Evol..

[61] O’Leary N.A., Wright M.W., Brister J.R., Ciufo S., Haddad D., McVeigh R., Rajput B., Robbertse B., Smith-White B., Ako-Adjei D. (2016). Reference sequence (RefSeq) database at NCBI: Current status, taxonomic expansion, and functional annotation. Nucleic Acids Res..

[62] Finn R.D., Coggill P., Eberhardt R.Y., Eddy S.R., Mistry J., Mitchell A.L., Potter S.C., Punta M., Qureshi M., Sangrador-Vegas A. (2016). The Pfam protein family’s database: Towards a more sustainable future. Nucleic Acids Res..

[63] Bairoch A., Apweiler R. (2000). The SWISS-PROT protein sequence database and its supplement TrEMBL in 2000. Nucleic Acids Res..

[64] Cantarel B.L., Coutinho P.M., Rancurel C., Bernard T., Lombard V., Henrissat B. (2009). The Carbohydrate-Active EnZymes database (CAZy): An expert resource for Glycogenomics. Nucleic Acids Res..

[65] Blin K., Shaw S., Kloosterman A.M., Charlop-Powers Z., van Wezel G.P., Medema M.H., Weber T. (2021). antiSMASH 6.0: Improving cluster detection and comparison capabilities. Nucleic Acids Res..

[66] Chaudhari N.M., Gupta V.K., Dutta C. (2016). BPGA- an ultra-fast pan-genome analysis pipeline. Sci. Rep..

[67] Schultzhaus Z., Chen A., Kim S., Shuryak I., Chang M., Wang Z. (2019). Transcriptomic analysis reveals the relationship of melanization to growth and resistance to gamma radiation in *Cryptococcus neoformans*. Environ. Microbiol..

[68] Molina-Menor E., Gimeno-Valero H., Pascual J., Peretó J., Porcar M. (2020). High Culturable Bacterial Diversity from a European Desert: The Tabernas Desert. Front. Microbiol..

[69] Kim M., Oh H.S., Park S.C., Chun J. (2014). Towards a taxonomic coherence between average nucleotide identity and 16S rRNA gene sequence similarity for species demarcation of prokaryotes. Int. J. Syst. Evol. Microbiol..

[70] Wayne L.G. (1988). International Committee on Systematic Bacteriology: Announcement of the report of the ad hoc Committee on Reconciliation of Approaches to Bacterial Systematics. Zentralblatt fur Bakteriologie, Mikrobiologie, und Hygiene. Zentralbl. Bakteriol. Mikrobiol. Hyg. Ser. A.

[71] Tatusov R.L., Natale D.A., Garkavtsev I.V., Tatusova T.A., Shankavaram U.T., Rao B.S., Kiryutin B., Galperin M.Y., Fedorova N.D., Koonin E.V. (2001). The COG database: New developments in phylogenetic classification of proteins from complete genomes. Nucleic Acids Res..

[72] Ye L.F., Chaudhary K.R., Zandkarimi F., Harken A.D., Kinslow C.J., Upadhyayula P.S., Dovas A., Higgins D.M., Tan H., Zhang Y. (2020). Radiation-Induced Lipid Peroxidation Triggers Ferroptosis and Synergizes with Ferroptosis Inducers. ACS Chem. Biol..

[73] Lin M.T., Beal M.F. (2006). Mitochondrial dysfunction and oxidative stress in neurodegenerative diseases. Nature.

[74] Ogwu M.C., Srinivasan S., Dong K., Ramasamy D., Waldman B., Adams J.M. (2019). Community Ecology of Deinococcus in Irradiated Soil. Microb. Ecol..

[75] Dalziel K., Egan R.R. (1972). The binding of oxidized coenzymes by glutamate dehydrogenase and the effects of glutarate and purine nucleotides. Biochem. J..

[76] Veech R.L., Todd King M., Pawlosky R., Kashiwaya Y., Bradshaw P.C., Curtis W. (2019). The "great" controlling nucleotide coenzymes. IUBMB Life.

[77] Tettelin H., Riley D., Cattuto C., Medini D. (2008). Comparative genomics: The bacterial pan-genome. Curr. Opin. Microbiol..

[78] Wang S., Jiang L., Hu Q., Cui L., Zhu B., Fu X., Lai Q., Shao Z., Yang S. (2021). Characterization of *Sulfurimonas hydrogeniphila* sp. nov., a Novel Bacterium Predominant in Deep-Sea Hydrothermal Vents and Comparative Genomic Analyses of the Genus Sulfurimonas. Front. Microbiol..

[79] Torres Manno M.A., Pizarro M.D., Prunello M., Magni C., Daurelio L.D., Espariz M. (2019). GeM-Pro: A tool for genome functional mining and microbial profiling. Appl. Microbiol. Biotechnol..

[80] Ivancic T., Jamnik P., Stopar D. (2013). Cold shock CspA and CspB protein production during periodic temperature cycling in Escherichia coli. BMC Res. Notes.

[81] Hakiem O.R., Parijat P., Tripathi P., Batra J.K. (2020). Mechanism of HrcA function in heat shock regulation in Mycobacterium tuberculosis. Biochimie.

[82] Morohoshi F., Munakata N. (1987). Multiple species of Bacillus subtilis DNA alkyltransferase involved in the adaptive response to simple alkylating agents. J. Bacteriol..

[83] Keck J.L., Goedken E.R., Marqusee S. (1998). Activation/attenuation model for RNase H. A one-metal mechanism with second-metal inhibition. J. Biol. Chem..

[84] Voloshin O.N., Vanevski F., Khil P.P., Camerini-Otero R.D. (2003). Characterization of the DNA damage-inducible helicase DinG from Escherichia coli. J. Biol. Chem..

[85] Arciszewska L.K., Sherratt D.J. (1995). Xer site-specific recombination in vitro. EMBO J..

[86] Barre F.X., Søballe B., Michel B., Aroyo M., Robertson M., Sherratt D. (2001). Circles: The replication-recombination-chromosome segregation connection. Proc. Natl. Acad. Sci. USA.

[87] Raman K., Yeturu K., Chandra N. (2008). targetTB: A target identification pipeline for Mycobacterium tuberculosis through an interactome, reactome and genome-scale structural analysis. BMC Syst. Biol..

[88] Gong C., Martins A., Bongiorno P., Glickman M., Shuman S. (2004). Biochemical and genetic analysis of the four DNA ligases of mycobacteria. J. Biol. Chem..

[89] Yeiser B., Pepper E.D., Goodman M.F., Finkel S.E. (2002). SOS-induced DNA polymerases enhance long-term survival and evolutionary fitness. Proc. Natl. Acad. Sci. USA.

[90] Huang Y.H., Huang C.Y. (2021). Comparing SSB-PriA Functional and Physical Interactions in Gram-Positive and -Negative Bacteria. Methods Mol. Biol..

[91] Slade D., Radman M. (2011). Oxidative stress resistance in *Deinococcus radiodurans*. Microbiol. Mol. Biol. Rev. MMBR.

[92] Pareek C.S., Smoczynski R., Tretyn A. (2011). Sequencing technologies and genome sequencing. J. Appl. Genet..

[93] Scutari M., Mackay I., Balding D. (2016). Using Genetic Distance to Infer the Accuracy of Genomic Prediction. PLoS Genet..

[94] Ríos-Touma B., Holzenthal R.W., Rázuri-Gonzales E., Heckenhauer J., Pauls S.U., Storer C.G., Frandsen P.B. (2022). De Novo Genome Assembly and Annotation of an Andean Caddisfly, *Atopsyche davidsoni* Sykora, 1991, a Model for Genome Research of High-Elevation Adaptations. Genome Biol. Evol..

[95] Cohan F.M., Perry E.B. (2007). A systematics for discovering the fundamental units of bacterial diversity. Curr. Biol. CB.

[96] Sonis S.T. (2021). Superoxide Dismutase as an Intervention for Radiation Therapy-Associated Toxicities: Review and Profile of Avasopasem Manganese as a Treatment Option for Radiation-Induced Mucositis. Drug Des. Dev. Ther..

[97] Liu J.F., Wang X., Tan H.N., Liu H., Wang Y.G., Chen R.Q., Cao J.C., Wang F.S. (2010). Effect of heparin-superoxide dismutase on γ-radiation induced DNA damage in vitro and in vivo. Drug Discov. Ther..

[98] Kıvrak E.G., Yurt K.K., Kaplan A.A., Alkan I., Altun G. (2017). Effects of electromagnetic fields exposure on the antioxidant defense system. J. Microsc. Ultrastruct..

[99] Lu J., Holmgren A. (2014). The thioredoxin antioxidant system. Free Radic. Biol. Med..

[100] Schlesinger D.J. (2007). Role of RecA in DNA damage repair in *Deinococcus radiodurans*. FEMS Microbiol. Lett..

[101] Smith J., Modrich P. (1996). Mutation detection with MutH, MutL, and MutS mismatch repair proteins. Proc. Natl. Acad. Sci. USA.

[102] Burghout P., Bootsma H.J., Kloosterman T.G., Bijlsma J.J., de Jongh C.E., Kuipers O.P., Hermans P.W. (2007). Search for genes essential for pneumococcal transformation: The RADA DNA repair protein plays a role in genomic recombination of donor DNA. J. Bacteriol..

[103] Saveson C.J., Lovett S.T. (1999). Tandem repeat recombination induced by replication fork defects in Escherichia coli requires a novel factor, RadC. Genetics.

[104] Feliciello I., Zahradka D., Zahradka K., Ivanković S., Puc N., Đermić D. (2018). RecF, UvrD, RecX and RecN proteins suppress DNA degradation at DNA double-strand breaks in *Escherichia coli*. Biochimie.

[105] Yang O., Ha T. (2018). Single-Molecule Studies of ssDNA-Binding Proteins Exchange. Methods Enzymol..

[106] Crowley D.J., Boubriak I., Berquist B.R., Clark M., Richard E., Sullivan L., DasSarma S., McCready S. (2006). The uvrA, uvrB and uvrC genes are required for repair of ultraviolet light induced DNA photoproducts in *Halobacterium* sp. NRC-1. Saline Syst..

[107] Norais C.A., Chitteni-Pattu S., Wood E.A., Inman R.B., Cox M.M. (2009). DdrB protein, an alternative *Deinococcus radiodurans* SSB induced by ionizing radiation. J. Biol. Chem..

[108] Zahradka K., Slade D., Bailone A., Sommer S., Averbeck D., Petranovic M., Lindner A.B., Radman M. (2006). Reassembly of shattered chromosomes in *Deinococcus radiodurans*. Nature.

[109] Repar J., Cvjetan S., Slade D., Radman M., Zahradka D., Zahradka K. (2010). RecA protein assures fidelity of DNA repair and genome stability in *Deinococcus radiodurans*. DNA Repair.

[110] Pellicanò G., Al Mamun M., Jurado-Santiago D., Villa-Hernández S., Yin X., Giannattasio M., Lanz M.C., Smolka M.B., Yeeles J., Shirahige K. (2021). Checkpoint-mediated DNA polymerase ε exonuclease activity curbing counteracts resection-driven fork collapse. Mol. Cell.

[111] Tanaka M., Narumi I., Funayama T., Kikuchi M., Watanabe H., Matsunaga T., Nikaido O., Yamamoto K. (2005). Characterization of Pathways Dependent on the uvsE, uvrA1, or uvrA2 Gene Product for UV Resistance in *Deinococcus radiodurans*. J. Bacteriol..

[112] Le S., Serrano E., Kawamura R., Carrasco B., Yan J., Alonso J.C. (2017). Bacillus subtilis RecA with DprA-SsbA antagonizes RecX function during natural transformation. Nucleic Acids Res..

[113] Grant R.A., Filman D.J., Finkel S.E., Kolter R., Hogle J.M. (1998). The crystal structure of Dps, a ferritin homolog that binds and protects DNA. Nat. Struct. Biol..

[114] Tseng C.C., Murni L., Han T.W., Arfiati D., Shih H.T., Hu S.Y. (2019). Molecular Characterization and Heterologous Production of the Bacteriocin Peocin, a DNA Starvation/Stationary Phase Protection Protein, from *Paenibacillus ehimensis* NPUST1. Molecules.

[115] Van Dyk T.K., DeRose E.J., Gonye G.E. (2001). LuxArray, a high-density, genomewide transcription analysis of Escherichia coli using bioluminescent reporter strains. J. Bacteriol..

[116] Kosinski J., Feder M., Bujnicki J.M. (2005). The PD-(D/E) XK superfamily revisited: Identification of new members among proteins involved in DNA metabolism and functional predictions for domains of (hitherto) unknown function. BMC Bioinform..

[117] Makharashvili N., Koroleva O., Bera S., Grandgenett D.P., Korolev S. (2004). A Novel Structure of DNA Repair Protein RecO from *Deinococcus radiodurans*. Structure.

[118] Wang Y., Branicky R., Noë A., Hekimi S. (2018). Superoxide dismutases: Dual roles in controlling ROS damage and regulating ROS signaling. Int. J. Biochem. Cell Biol..

[119] Rattanawong K., Koiso N., Toda E., Kinoshita A., Tanaka M., Tsuji H., Okamoto T. (2021). Regulatory functions of ROS dynamics via glutathione metabolism and glutathione peroxidase activity in developing rice zygote. Plant J. Cell Mol. Biol..

[120] Kocsy G., Galiba G., Brunold C. (2001). Role of glutathione in adaptation and signalling during chilling and cold acclimation in plants. Physiol. Plant..

[121] Daly M.J. (2009). A new perspective on radiation resistance based on *Deinococcus radiodurans*. Nat. Rev. Microbiol..

[122] Xu Z., Tian B., Sun Z., Lin J., Hua Y. (2007). Identification and functional analysis of a phytoene desaturase gene from the extremely radioresistant bacterium *Deinococcus radiodurans*. Microbiology.

[123] Davydova O.K., Deriabin D.G., El’-Registan G.I. (2006). Influence of chemical analogues of microbial autoregulators on the sensitivity of DNA to UV radiation. Mikrobiologiia.

