# Supplementary material for: Genomic Insights into the Radiation-Resistant Capability of Sphingomonas qomolangmaensis S5-59T and Sphingomonas glaciei S8-45T, Two Novel Bacteria from the North Slope of Mount Everest
Source: Microorganisms. 2022 Oct 14;10(10):2037. doi: 10.3390/microorganisms10102037 (PMC9611098; doi:10.3390/microorganisms10102037)
Supplement: Supplementary file 1 [file microorganisms-10-02037-s001.zip › Supplymentary Materials.pdf]

Supplementary Materials

Figures

Figures S1-S7

Tables S1-S8

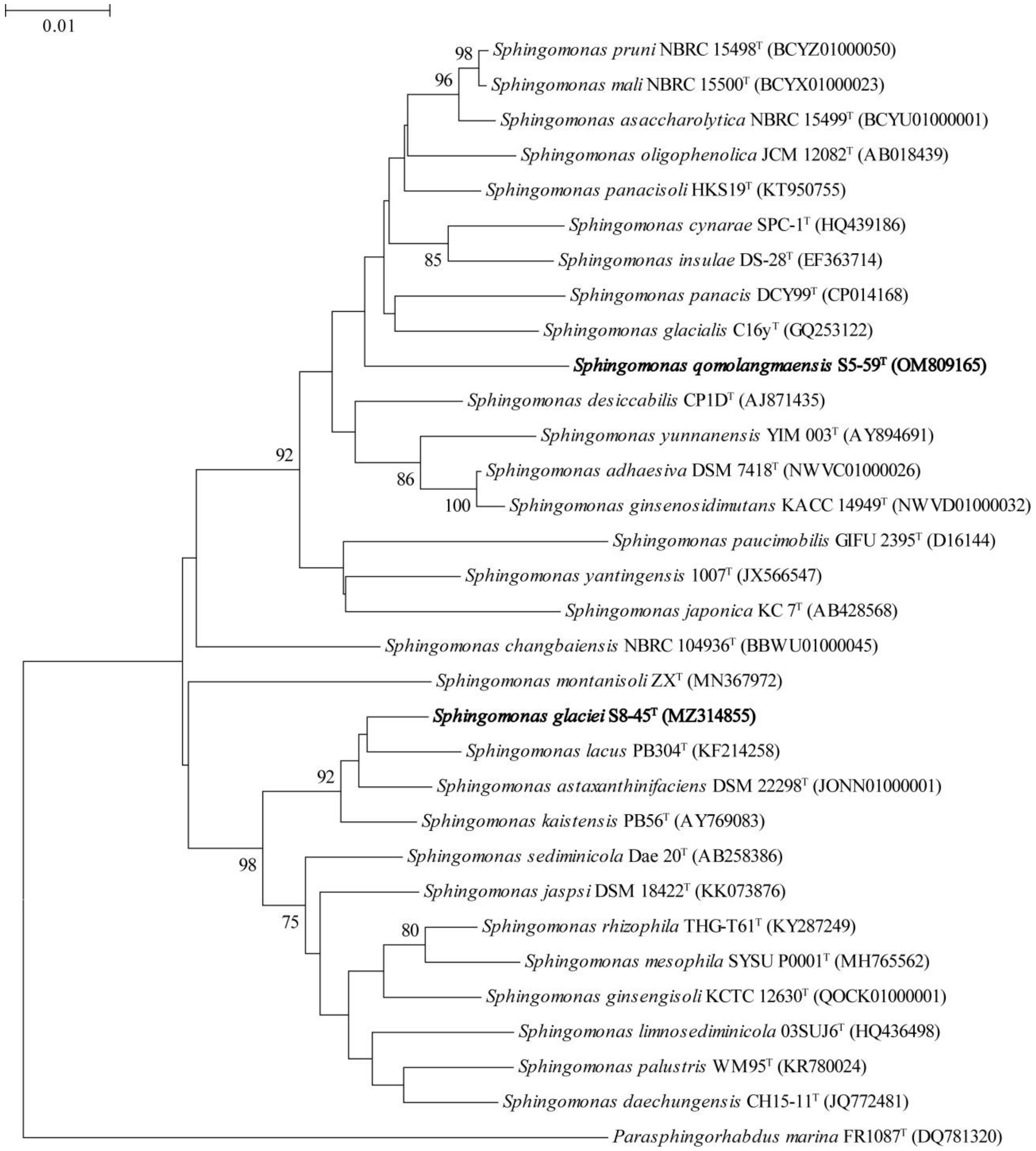

Figure S1 Minimum-evolution phylogenetic tree based on 16S rRNA gene sequences of the strain S5-59^T, S8-45^T, and the type strains of other closely related species in the genus *Sphingomonas* and *Parasphingorhabdus*. *Parasphingorhabdus marina* FR1087^T (DQ781320) was used as an outgroup. The numbers on the tree indicate the percentages of bootstrap sampling derived from 1000 replications and the bootstrap values higher than 70% are shown. Bar, 0.01 substitutions per nucleotide position.

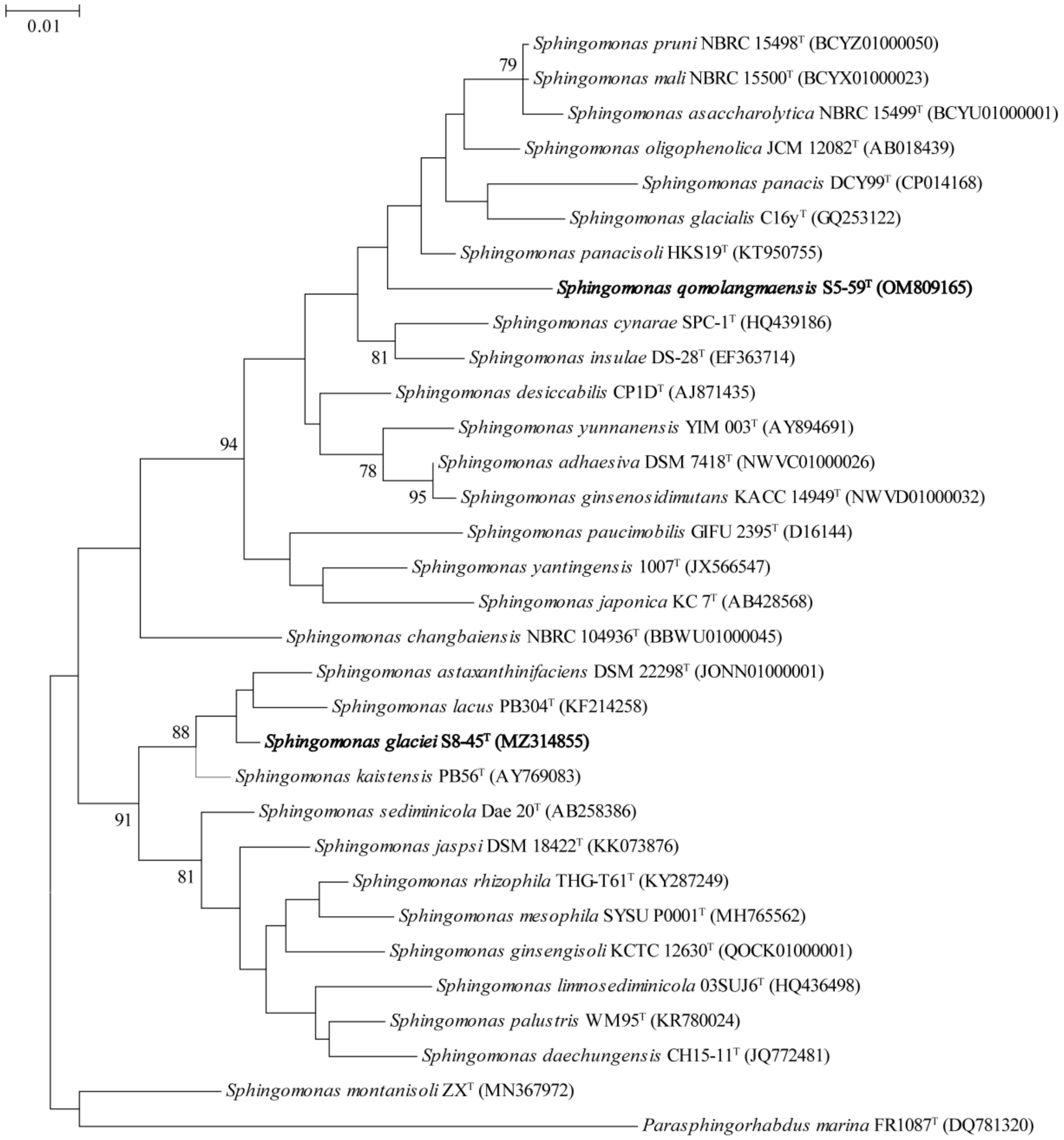

Figure S2 Maximum-likelihood phylogenetic tree based on 16S rRNA gene sequences of the strain S5-59^T, S8-45^T, and the type strains of other closely related species in the genus *Sphingomonas* and *Parasphingorhabdus*. *Parasphingorhabdus marina* FR1087^T (DQ781320) was used as an outgroup. The numbers on the tree indicate the percentages of bootstrap sampling derived from 1000 replications and the bootstrap values higher than 70% are shown. Bar, 0.01 substitutions per nucleotide position.

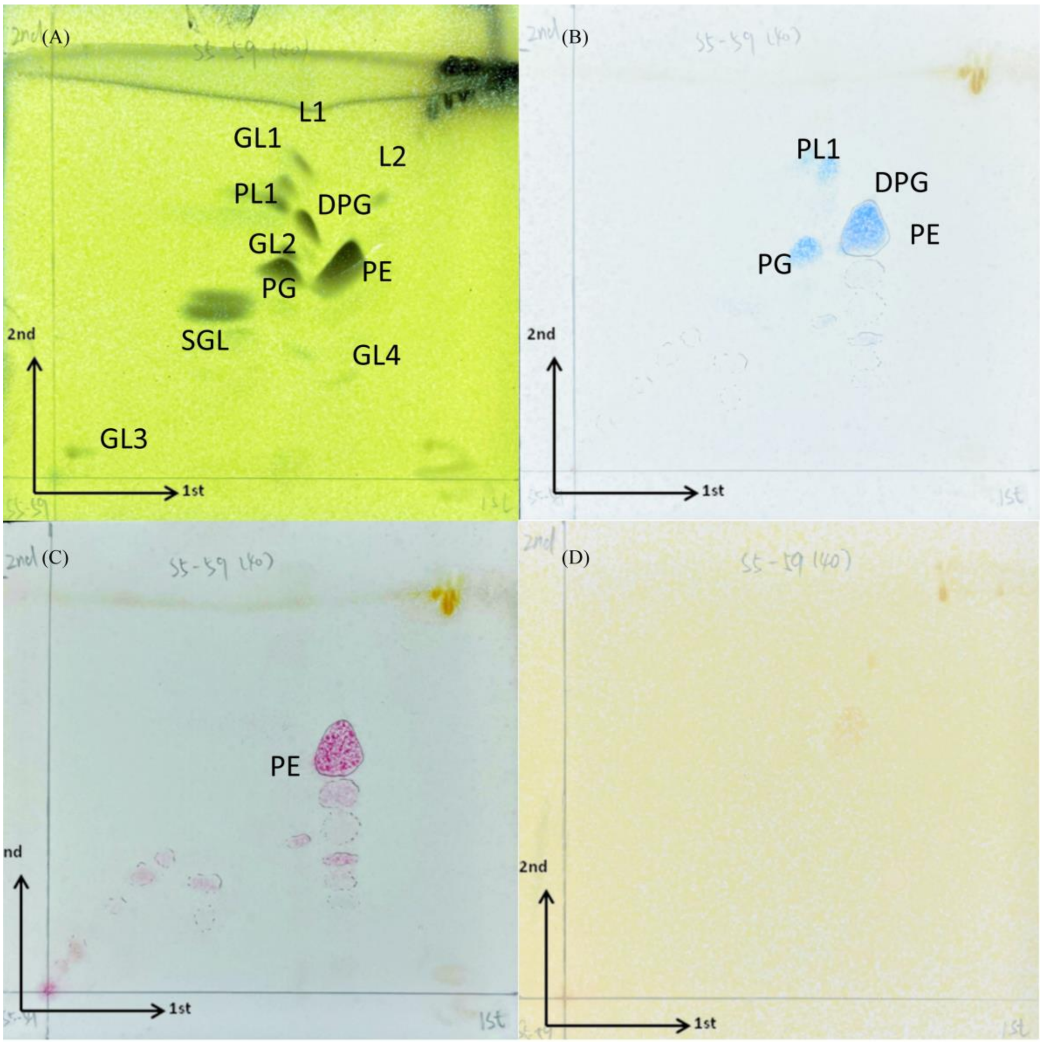

Figure S3a Polar lipids profile of strain S5-59^T. Total lipids were visualized after two-dimensional TLC and applying 5% ethanolic molybdatophosphoric acid. The solvent system was phosphomolybdic acid (**A**), molybdenum blue (**B**), indigohydrone (**C**), and α-naphthol (**D**) from left to right and top to bottom.

Abbreviations: DPG, diphosphatidylglycerol; PG, phosphatidylglycerol; PE, phosphatidylethanolamine; PL1, unidentified phospholipid; GL1-4, unidentified glycolipids;SGL, sphingoglycolipid; L1-2,unidentified lipid .

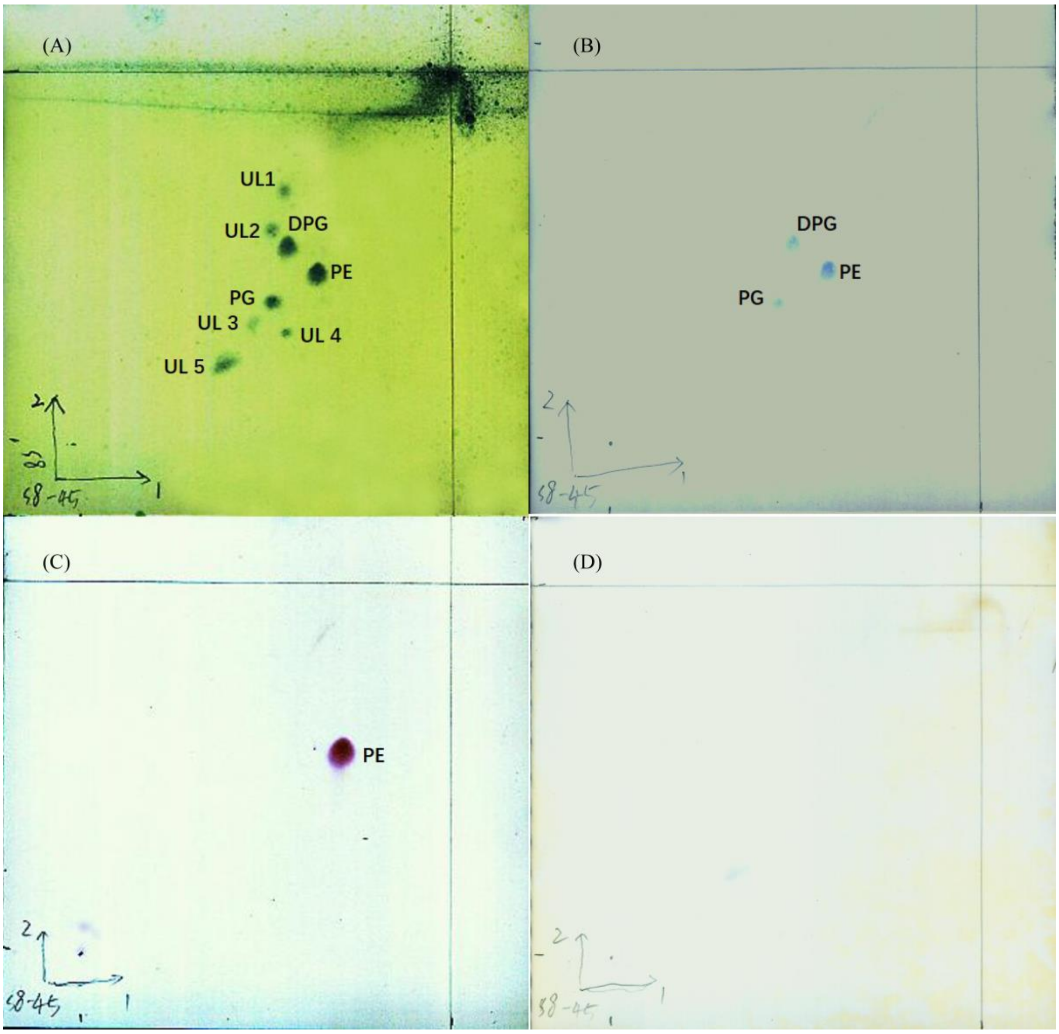

Figure S3b Polar lipids profile of strain S8-45[T]. Total lipids were visualized after two-dimensional TLC and applying 5% ethanolic molybdatophosphoric acid. The solvent system was phosphomolybdic acid (**A**), molybdenum blue (**B**), indigohydrone (**C**), and α-naphthol (**D**) from left to right and top to bottom.

Abbreviations: DPG, diphosphatidyl glycerol; PG, phosphatidyl glycerol; PE, phosphatidyl ethanolamine; UL 1-5, unidentified lipids.

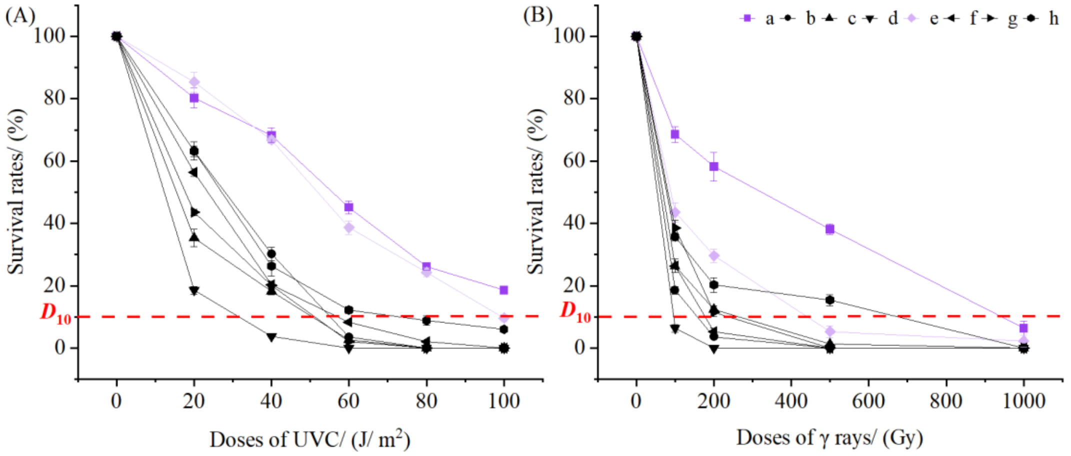

Figure S4 The survival rates of strains S5-59^T, S8-45^T and reference strains after irradiation with different dose of UVC (A) and γ rays' (B) radiation. D_{10} – values were displayed by red dash line. a S5-59^T; b *S. panacisoli* HKS19^T; c *S. asaccharolytica* NBRC 15499^T; d *S. panacis* DCY99^T; e S8-45^T; f *S. kaistensis* PB56^T; g *S. astaxanthinifaciens* DSM 22298^T and h *S. ginsengisoli* KCTC 12630^T.

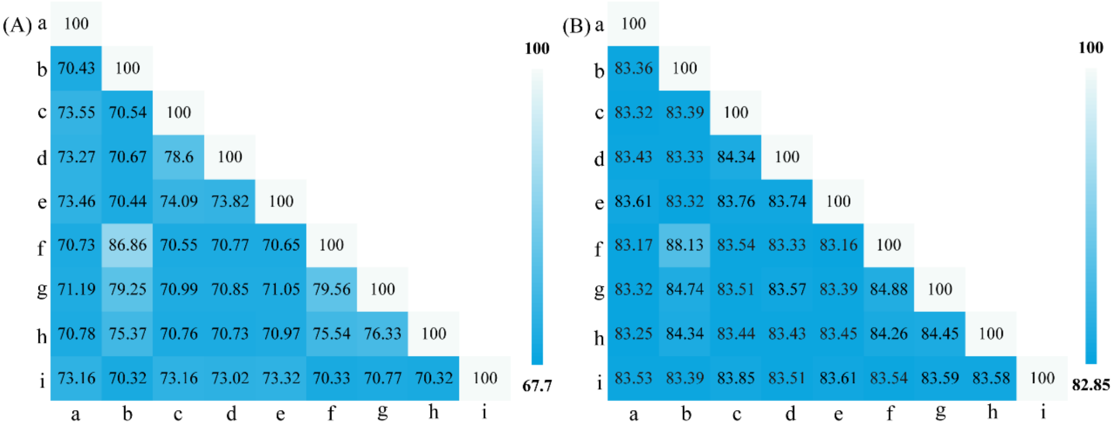

Figure S5 Genome comparisons of strains S5-59^T, and S8-45^T and their related reference strains including ANIb value (A) and ANIm value (B). a-h represented S5-59^T, S8-45^T, *S. panacisoli* HKS19^T, *S. asaccharolytica* NBRC 15499^T, *S. panacis* DCY99^T, *S. kaistensis* PB56^T, *S. astaxanthinifaciens* DSM 22298^T and *S. ginsengisoli* KCTC 12630^T respectively. i was the type species of *Sphingomonas*, *S. paucimobilis* DSM 1098 ^{T}.

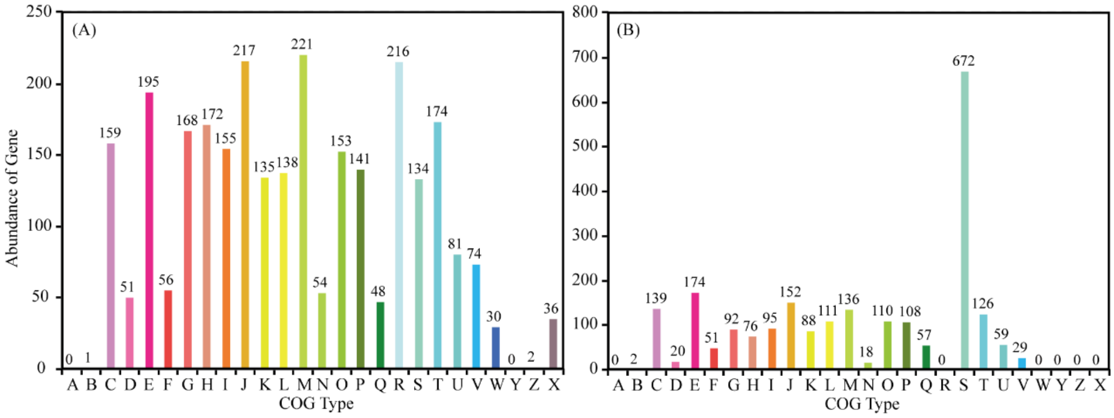

Figure S6 COG functional categories of the strains S5-59^{T} (A) and S8-45^{T} (B) genomes. The description of COG type was shown in Table S3.

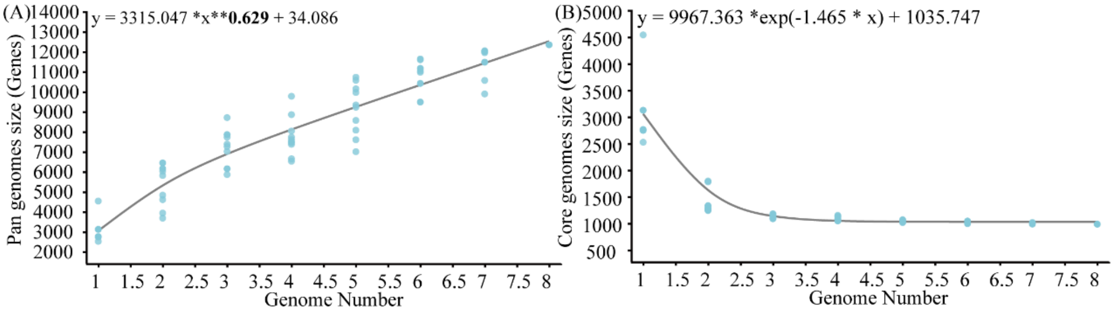

Figure S7 Characteristic curves of the pan-genome and core genome of S5-59^{T} (A) and S8-45^{T} (B).

Tables

Table S1. Whole cellular fatty acids composition of S5-59^T and the closely related type strains of the genus *Sphingomonas*.

Fatty acids (%)	1	2	3	4	5	6	7	8
Saturated fatty acids								
$C_{16:0}$	4.2	6.7	5.8	13.0	4.0	4.7	10.1	15.1
$C_{18:0}$	0.5	L	ND	ND	0.5	L	L	1.8
$C_{17:0}$	L	0.9	ND	ND	L	1.4	0.9	2.0
Unsaturated fatty acids								
$C_{16:1}\ \omega 5c$	0.7	1.4	L	ND	3.6	2.0	ND	ND
$C_{17:1}\ \omega 6c$	2.8	3.3	0.6	ND	7.1	13.7	9.2	12.8
$C_{17:1}\ \omega 8c$	L	L	ND	ND	0.7	3.1	1.3	2.1
$C_{18:1}\ \omega 5c$	0.5	L	ND	ND	0.6	1.1	0.8	1.4
$C_{18:1}\ \omega 7c_{\text{11-methyl}}$	ND	ND	45.5	7.2	2.9	2.1	3.0	ND
Hydroxy fatty acids								
$C_{17:0}$ 2OH	ND	L	ND	ND	0.8	13.8	ND	ND
$C_{18:1\ iso}$ 2OH	L	L	ND	ND	ND	2.0	1.5	ND
Summed Feature 3	37.4	28.7	22.4	2.1	42.4	23.2	27.6	27.3
Summed Feature 8	44.9	42.4	36.6	55.9	42.0	31.1	42.0	46.9

Strains: 1. S5-59^T; 2. *S. panacisoli* HKS19^T; 3. *S. asaccharolytica* DSM10564^T; 4. *S. panacis* DCY99^T; 5. S8-45^T; 6. *S. kaistensis* PB56^T; 7. *S. astaxanthinifaciens* DSM 22298^T; 8. *S. ginsengisoli* KCTC 12630^T. Only fatty acids percentages amounting to 0.5% or higher were shown. L (<0.5%); ND, not detected. All data were obtained in this study.

Summed features represent groups of two fatty acids that could not be separated by GLC and the MIDI system. Summed feature 3 contained 16:1 $\omega 6c$ and / or 16:1 $\omega 7c$, and summed feature 8 contained 18:1 $\omega 6c$ and /or 18:1 $\omega 7c$.

Table S2. General genomic characteristics comparison of strains S5-59^T and S8-45^T with closely related type strains.

Characteristics	1	2	3	4	5	6	7	8
Sequence status	Complete	Complete	Draft	Complete	Complete	Complete	Complete	Complete
Genome size (bp)	3429145	3403025	4608803	5322941	2880162	2846238	2533034	3037290
Contig N50	3429145	3403025	50453	5003808	2880162	2846238	2144484	3037290
CDS	3213	3473	4682	5110	2813	2799	2562	3022
RNA	60	51	52	68	53	52	52	54
rRNA	6	3	3	9	3	3	3	3
tRNA	48	45	46	50	47	46	46	48
5S rRNA	2	1	1	3	1	1	1	1
16S rRNA	2	1	1	3	1	1	1	1
23S rRNA	2	1	1	3	1	1	1	1
GI	6	4	3	17	6	6	4	8
KEGG	1763	1558	1988	2226	1424	1526	1452	885
COG	2546	2581	2935	3882	2273	2157	1987	2239
NR	3076	3354	3854	4840	2811	2754	2493	2877
Pfam	2617	2667	3508	3992	2271	2255	2075	2344
GO	1379	1879	2655	3459	574	922		1059
Swiss-Prot	2147	2150	2864	3318	1874	1855	1708	1917
CAZyme	129	92	189	216	76	80	63	123
Secondary Metabolite	6	3	7	4	2	2	2	3
DNA G+C content (mol%)	66.4	65.1	64.8	64.4	66.6	69.9	63.3	66.8
GenBank accession number	GCA_024496245.1	GCA_007859635.1	GCA_001598355.1	GCA_001717955.1	GCA_023380025.1	GCA_011927725.1	GCA_000711715.1	GCA_009363895.1

Strains: 1. S5-59^T; 2. *S. panacisoli* HKS19^T; 3. *S. asaccharolytica* NBRC 15499^T; 4. *S. panacis* DCY99^T; 5. S8-45^T; 6. *S.kaistensis* PB56^T; 7. *S. astaxanthinifaciens* DSM 22298^T; 8. *S. ginsengisoli* KCTC 12630^T.

Table S3 The description of COG type.

COG Type	Type Description	Category
**A**	RNA processing and modification	INFORMATION STORAGE AND PROCESSING
**B**	Chromatin structure and dynamics	
**C**	Energy production and conversion	METABOLISM
**D**	Cell cycle control, cell division, chromosome partitioning	CELLULAR PROCESSES AND SIGNALING
**E**	Amino acid transport and metabolism	METABOLISM
**F**	Nucleotide transport and metabolism	
**G**	Carbohydrate transport and metabolism	
**H**	Coenzyme transport and metabolism	
**I**	Lipid transport and metabolism	
**J**	Translation, ribosomal structure and biogenesis	INFORMATION STORAGE AND PROCESSING
**K**	Transcription	
**L**	Replication, recombination and repair	
**M**	Cell wall/membrane/envelope biogenesis	CELLULAR PROCESSES AND SIGNALING
**N**	Cell motility	
**O**	Posttranslational modification, protein turnover, chaperones	
**P**	Inorganic ion transport and metabolism	METABOLISM
**Q**	Secondary metabolites biosynthesis, transport and catabolism	
**R**	General function prediction only	POORLY CHARACTERIZED
**S**	Function unknown	
**T**	Signal transduction mechanisms	CELLULAR PROCESSES AND SIGNALING
**U**	Intracellular trafficking, secretion, and vesicular transport	
**V**	Defense mechanisms	
**W**	Extracellular structures	
**Z**	Cytoskeleton	
**X**	Mobilome: prophages, transposons	

Table S4 Partial unique genes of pan-genome in strain S5-59^T.

Functional Description	Protein Description	GenBank (NCBI)	Protein Name
Replication, recombination and repair	ATP-dependent helicase YprA, contains C-terminal metal-binding DUF1998 domain	UUL83599	ATP-dependent DNA helicase RecG
		UUL81970	ATP-dependent DNA helicase
		UUL82086	ATP-dependent helicase HrpB
		UUL82530	ATP-dependent helicase
	3-methyladenine DNA glycosylase Mpg	UUL82206	DNA-3-methyladenine glycosylase 2 family protein
		UUL83160	DNA-3-methyladenine glycosylase
	RecA-family ATPase	UUL84166	recombinase RecA
Cell cycle control, cell division, chromosome partitioning	Spore cortex hydrolase SleL/YaaH, N-acetylglucosaminidase of GH18 family	UUL81304	bifunctional GNAT family N-acetyltransferase/carbon-nitrogen hydrolase family protein
Defense mechanisms	VapC family ribonuclease, toxin component of the VapBC toxin-antitoxin module, contains PIN domain	UUL81688	type II toxin-antitoxin system VapC family toxin
		UUL81737	
		UUL82936	
		UUL83370	
		UUL83620	
		UUL83627	
	mRNA-degrading endonuclease RelE, toxin component of the RelBE toxin-antitoxin system	UUL81874	type II toxin-antitoxin system RelE/ParE family toxin
		UUL81892	
		UUL82885	
		UUL82975	
	Toxin component of the Txe-Axe toxin-antitoxin module, Txe/YoeB family	UUL81894	Txe/YoeB family addiction module toxin
	Spore cortex hydrolase SleL/YaaH, N-acetylglucosaminidase of GH18 family	UUL81304	bifunctional GNAT family N-acetyltransferase/carbon-nitrogen hydrolase family protein
	Sulfur carrier protein TusA (tRNA thiolation, molybdenum cofactor biosynthesis)	UUL81244	sulfurtransferase TusA family protein
	Cbb3-type cytochrome oxidase, cytochrome c subunit FixO	UUL84046	cytochrome-c oxidase, cbb3-type subunit III
		UUL84048	cytochrome-c oxidase, cbb3-type subunit II
	Cbb3-type cytochrome oxidase, subunit 1	UUL84222	cytochrome-c oxidase, cbb3-type subunit I
	Cbb3-type cytochrome oxidase, subunit 3	UUL84047	cbb3-type cytochrome c oxidase subunit 3
	Nitrogen fixation protein FixH	UUL84044	FixH family protein
	Bacteriorhodopsin	UUL81543	bacteriorhodopsin-like
General function prediction only	Predicted ATPase, archaeal AAA+ ATPase superfamily	UUL81953	CDC48 family AAA ATPase
		UUL84243	YifB family Mg chelatase-like AAA ATPase
	Predicted NAD/FAD-dependent oxidoreductase	UUL82862<br>UUL83726<br>UUL83692<br>UUL83360	SDR family NAD(P)-dependent oxidoreductase

		UUL83092<br>UUL83629<br>UUL83668<br>UUL83844<br>UUL81980	NAD(P)/FAD-dependent oxidoreductase
**Function unknown**	Uncharacterized membrane protein YbaN, DUF454 family	UUL84293	YbaN family protein
	Uncharacterized short protein YbdD, DUF466 family	UUL81904	YbdD/YjiX family protein
	Uncharacterized conserved protein YcfJ, contains glycine zipper 2TM domain	UUL81328<br>UUL81383<br>UUL81564	glycine zipper 2TM domain-containing protein
	Uncharacterized membrane protein YagU, involved in acid resistance, DUF1440 family	UUL83289	DUF1440 domain-containing protein

Table S5 The specific genes of pan-genome in strain S8-45^{T}.

**Functional Description**	**Protein Description**	**GenBank (NCBI)**	**Protein Name**
Cell wall/membrane/envelope biogenesis	Membrane-bound lytic murein transglycosylase MltF	UUR08537	lytic murein transglycosylase
	Exopolysaccharide synthesis protein ExoD	UUR06960	exopolysaccharide biosynthesis polyprenyl glycosylphosphotransferase
Defense mechanisms	Predicted type IV restriction endonuclease	UUR08283	DNA mismatch endonuclease Vsr
	Predicted restriction endonuclease	UUR07574	restriction endonuclease
General function prediction only	Predicted nuclease, RecB family	UUR07883	RecX family transcriptional regulator
	Predicted RNA-binding protein, contains PUA-like EVE domain	UUR07764	tRNA-binding protein
Inorganic ion transport and metabolism	iron-containing redox enzyme family protein	UUR08173	iron-containing redox enzyme family protein
	Intracellular sulfur oxidation protein, DsrE/DsrF family	UUR07956	ferredoxin:protochlorophyllide reductase (ATP-dependent) iron-sulfur ATP-binding protein
	Periplasmic deferrochelatase/peroxidase EfeB	UUR06761	catalase/peroxidase HPI

Table S6 Features of the GIs found in the genome of *S. qomolangmaensis* S5-59^T.

GI	Length	Total no. of gene	hypothetical proteins	Predicted function
1	114651	97	20	Amino acid transport and metabolism/Signaling and cellular processes/Metabolism /DNA repair
2	85514	67	2	Amino acid metabolism/Signaling and cellular processes/Carbohydrate transport and metabolism/Membrane transport/DNA repair
3	51352	39	10	Carbohydrate transport and metabolism /Transcription factors/ Posttranslational modification
4	20001	23	5	Amino acid metabolism/ Posttranslational modification
5	19078	23	5	Amino acid metabolism/ Membrane/envelope biogenesis
6	16348	19	9	Amino acid metabolism
7	16036	18	2	Lipid transport and metabolism/ Amino acid metabolism/ Carbohydrate transport and metabolism
8	11928	13	4	Amino acid metabolism/ Peptidases and inhibitors/ Posttranslational modification
9	8907	8	0	Metabolism/ Defense mechanisms / DNA repair
Total	343815	307	57	

Table S7 Features of the GIs found in the genome of *S. glaciei* S8-45^T.

GI	Length	Total no. of gene	hypothetical proteins	Predicted function
1	34165	30	9	Membrane transport /Amino acid transport and metabolism/ /Metabolism /DNA repair
2	27938	25	6	Amino acid metabolism/ Carbohydrate transport and metabolism/Membrane transport/DNA repair
3	26855	28	5	Transcription factors/ Posttranslational modification/ DNA repair
4	20856	23	5	Posttranslational modification/ Energy production and conversion
5	18840	20	6	Amino acid metabolism/ Membrane/ Posttranslational modification
6	2716	7	2	Membrane/envelope biogenesis
Total	131370	133	33	

Table S8 List of partial genes encoding enzymatic activity antioxidant proteins involved in radiation-induced oxidative stress in the genomes of type strains S5-59^T and S8-45^T.

GenBank ID of S5-59^T	Gene Symbol	Description	GenBank ID of S8-45^T	Gene Symbol	Description
**UUL83639**		superoxide dismutase	**UUR08565**	-	superoxide dismutase
**UUL83875**		superoxide dismutase family protein	**UUR08996**	-	superoxide dismutase family protein
**UUL83714**		catalase	**UUR06761**	*katG*	catalase/peroxidase HPI
**UUL82868**	*katG*	catalase/peroxidase HPI	**UUR08815**		glutathione S-transferase
**UUL83417**		glutathione peroxidase	**UUR09149**	*gshB*	glutathione synthase
**UUL83536**<br>**UUL83537**<br>**UUL81731**		glutathione S-transferase	**UUR08404**<br>**UUR09367**	-	glutathione-dependent formaldehyde dehydrogenase
**UUL82894**<br>**UUL82958**<br>**UUL83036**<br>**UUL82605**		glutathione S-transferase family protein	**UUR09164**	*gorA*	glutathione-disulfide reductase
**UUL82748**		glutathione S-transferase N-terminal domain-containing protein	**UUR08814**	-	aldo/keto reductase family oxidoreductase
**UUL81655**	*gshB*	glutathione synthase	**UUR06881**	-	Gfo/Idh/MocA family oxidoreductase
**UUL81740**	*yghU*	glutathione-dependent disulfide-bond oxidoreductase	**UUR08173**	-	iron-containing redox enzyme family protein
**UUL83359**		glutathione-dependent formaldehyde dehydrogenase	**UUR09244**	*ychF*	redox-regulated ATPase YchF
**UUL83359**	*gorA*	glutathione-disulfide reductase	**UUR08219**	*msrB*	peptide-methionine (R)-S-oxide reductase MsrB
**UUL83278**	*ahpC*	alkyl hydroperoxide reductase subunit C	**UUR08218**	*msrA*	peptide-methionine (S)-S-oxide reductase MsrA
**UUL83279**	*ahpF*	alkyl hydroperoxide reductase subunit F	**UUR09377**<br>**UUR08986**<br>**UUR06751**	-	SDR family NAD(P)-dependent oxidoreductase
**UUL81740**	*yghU*	glutathione-dependent disulfide-bond oxidoreductase	**UUR08119**<br>**UUR08418**<br>**UUR08500**<br>**UUR08747**<br>**UUR09000**<br>**UUR09186**<br>**UUR06798**<br>**UUR06799**<br>**UUR06977**<br>**UUR07274**<br>**UUR07336**<br>**UUR07393**<br>**UUR09479**<br>**UUR07876**	-	SDR family oxidoreductase
**UUL81361**<br>**UUL81948**	*msrB*	peptide-methionine (R)-S-oxide reductase MsrB	**UUR08513**	*trxA*	thioredoxin TrxA
**UUL82677**<br>**UUL81959**	*msrA*	peptide-methionine (S)-S-oxide reductase MsrA	**UUR08266**	*trxB*	thioredoxin-disulfide reductase
**UUL81408**	*ychF*	redox-regulated ATPase YchF			
**UUL82862**<br>**UUL83360**<br>**UUL83692**	-	SDR family NAD(P)-dependent oxidoreductase			
**UUL83726**<br>**UUL82750**<br>**UUL84181**<br>**UUL83618**<br>**UUL83618**<br>**UUL83618**<br>**UUL83618**<br>**UUL84230**<br>**UUL84230**<br>**UUL84230**<br>**UUL84230**<br>**UUL84230**	-	SDR family oxidoreductase			
**UUL83766**	-	thiol-disulfide oxidoreductase DCC family protein			
**UUL82209**	*trxB*	thioredoxin-disulfide reductase			
**UUL83609**	*trxA*	thioredoxin TrxA			
